# Development of a Bioinspired Soft Robotic System for Teleoperated Endoscopic Surgery

**DOI:** 10.34133/cbsystems.0289

**Published:** 2025-06-12

**Authors:** Kefan Zhu, Chi Cong Nguyen, Bibhu Sharma, Phuoc Thien Phan, Trung Thien Hoang, James Davies, Adrienne Ji, Emanuele Nicotra, Jingjing Wan, Patrick Pruscino, Sumeet Walia, Tat Thang Vo-Doan, Soo Jay Phee, Shing Wong, Nigel H. Lovell, Thanh Nho Do

**Affiliations:** ^1^ Graduate School of Biomedical Engineering, UNSW, Kensington Campus, Sydney, NSW 2052, Australia.; ^2^ Centre for Opto-electronic Materials and Sensors, School of Engineering, RMIT University, Melbourne, VIC 3000, Australia.; ^3^ School of Mechanical and Mining Engineering, The University of Queensland, St Lucia, QLD 4072, Australia.; ^4^ School of Mechanical and Aerospace Engineering, Nanyang Technological University, Singapore 639798, Singapore.; ^5^ School of Clinical Medicine, UNSW Sydney and Prince of Wales Hospital, Randwick, Sydney, NSW 2031, Australia.; ^6^ Tyree Institute of Health Engineering (IHealthE), Faculty of Engineering, UNSW, Sydney, NSW 2052, Australia.

## Abstract

Endoscopic submucosal dissection (ESD) has emerged as a critical alternative to laparoscopic excisional surgery for the removal of early gastrointestinal tumors. However, current robotic systems for ESD face challenges with accessibility, dexterity, and precision in confined spaces due to limitations in actuation methods and mechanical design. To overcome these issues, a new motorless, master–slave soft robotic system using hydraulic actuation is introduced for ESD procedures. This system features dual soft robotic arms: one serves as an electrosurgical tool, and the other serves as a 3-jaw soft tubular grasper. Notably, the entire system is powered purely by hydraulic force, eliminating the need for DC motors or complex electronic controllers. Inspired by nature, the grasper ensures even force distribution and removes rotational motion, reducing the risk of iatrogenic injury. Its scalable design and compliant properties allow for effective tissue manipulation in tight spaces, with strong pulling forces generated by the embedded soft actuation network. In vitro and ex vivo experiments on fresh porcine tissues demonstrate the system’s ability to grip and perform electrosurgical cutting on simulated lesions. This innovation has the potential to be applied in other areas of endoscopic surgery as well.

## Introduction

Colorectal cancer (CRC) ranks as the third most diagnosed cancer globally and poses a significant mortality risk [1]. The 2 primary methods for removing CRC lesions from the colon include laparoscopic surgery (LS) and natural orifice transluminal endoscopic surgery (NOTES) [2,3]. LS needs small incisions to insert the surgical instruments into the surgical target by laparoscopes [4,5]. Although many surgical tools, including surgical graspers, have been developed to assist LS, such as Endowrist employed in the da Vinci system (Intuitive Surgical Inc., California, USA) or conventional grippers with flexible robotic arms [6–9], they cannot avoid this problem because of its rigidity [10,11].

With the increasing popularity of flexible endoscopes and surgical robots, NOTES takes the principles of minimal invasiveness further by utilizing endoscopes to access internal organs through the human body’s natural orifices without a laparoscope or external skin incisions allowing surgery on submucosal or extraluminal lesions [12,13]. NOTES represent an advanced form of LS by impeccably adhering to the principles of minimal invasiveness by avoiding external incision-related complications [14,15]. Endoscopic submucosal dissection (ESD) is the most common approach for treating early-stage CRC in NOTES, which is a technique used to remove precancerous lesions and other abnormal tissues from the gastrointestinal (GI) tract [16]. Current ESD systems rely on cable-driven mechanisms to actuate flexible robotic arms, which provide precise steering and high degrees of freedom (DOFs) for completing surgery. These robotic arms, exemplified by platforms like the micro-IGES system and the second-generation STRAS system (Strasbourg, France), are typically equipped with interchangeable tools such as electrocautery knives and graspers [17]. Grasper consists of 2 components: the manipulator and the gripping end-effector. The manipulator’s rotational and deflective capabilities enable safe navigation to the target plane, minimizing perforation risks, while the end-effector’s precision ensures controlled tissue interaction. In benchtop validation studies, the micro-IGES platform demonstrated excellent targeting accuracy (<1 mm spatial deviation) via antagonistic tendon-driven actuation. Its 7-DOF joints can generate 3.5 N of tissue manipulation force while maintaining instrument triangulation within a constrained rectal workspace of 40 mm diameter and 200 mm depth [18]. The STRAS platform has an additional 3 DOF compared to IGES with similar submillimeter accuracy, enabling advanced endoluminal procedures such as suturing and lesion dissection [19,20]. However, cable-driven systems face several fundamental drawbacks. Over longer paths, friction and hysteresis can introduce force transfer errors and reduce control accuracy, especially when navigating narrow regions of the descending colon [21–23]. Furthermore, reliance on microcontrollers, DC motors, and pumps can make these systems bulky, expensive, and more complex to operate—factors that limit their appeal for everyday clinical use [24].

In attempts to perform the ESD procedure throughout the entire colon, many low-friction actuation methods such as pneumatics [25], hydraulics [24,26], magnetics [27,28], and hybrid approaches [29] have been proposed. Magnetic actuators have the advantage of relying on non-contact force-generated wireless motion control, but their insufficient force generation, especially for tissue manipulation, makes them more suitable for applications within blood vessels. Furthermore, reliance on external magnetic fields compromises surgical stability [30]. Although pneumatic and hydraulic are both fluid drives, which have high compliance and frictionless force transmission, Yu et al. [31] demonstrated that the 2 drive modes have their focuses when driving similar devices at the same time. Due to its incompressibility, the hydraulic drive can achieve smoother movement, higher position control accuracy, and better resistance to force interference. Pneumatic devices show faster dynamics at relatively low pressures and are easier to maintain. Moreover, Focchi et al. [32] found that hydraulic drive has higher response frequency and faster response time when using the same McKibben muscle. Meller et al. [33] conducted energy conversion experiments for hydraulic and pneumatic operation in artificial muscles at 3.4 bar. The energy conversion efficiency of hydraulic operation was 53.3%, more than twice that of pneumatic operation at 19.1%. In contrast, hydraulic actuators are more suitable for ESD surgical environments due to their stability, precise control, fast response, and potential for miniaturization [29,34]. Beyond colorectal ESD, frictionless force transmission, miniaturization potential, and high compliance of hydraulic actuators enable navigation of irregular luminal structures and articulation exceeding 90°. For example, the surgical path from the nostril to the target in transnasal endoscopic surgery is always narrow and constrained [35]. Cable-driven systems struggle due to rigid components and limited force transmission, whereas hydraulic actuators enable smooth, compliant movement [36]. Similarly, in transurethral resection of bladder tumor, accessing the bladder neck—a constricted region at the bladder–urethral junction—demands transurethral insertion followed by retroflection maneuvers (bending angles of 90° to 180°) to position the end-effector to reach the lesion area around the bladder neck [37]. Cable-driven surgical systems face size limitations in procedures requiring articulation exceeding 90° to navigate irregular luminal anatomies [38].

Despite the advantages of hydraulic actuation in terms of maneuverability, tissue-adaptable gripping end-effectors remain essential for precise tissue manipulation. Most current end-effectors rely on 2-jaw grippers [39–41], which often require a rotational motion for effective tissue engagement [23] and with securely grasping cylindrical or spherical objects [42]. By comparison, 3-jaw mechanisms provide independent benefits, including more precise centering, greater anchoring force, and improved stability through uniform load distribution and increased contact area [43]. The additional jaw can increase the contact area with delicate or irregular tissues, effectively minimizing slippage and tissue damage while enabling more delicate, controlled manipulation. These attributes position 3-jaw grippers as a promising solution for complex surgical procedures demanding both precision and adaptability. While 3-jaw grippers are currently prioritized for organ manipulation, their mechanical advantages are demonstrated in laparoscopic tools, suggesting strong potential for ESD [44]. For example, Oshima et al. [45] designed a 15-mm-diameter 3-fingered robotic grasper with force distribution capabilities for LS. The MUltifunctional Smart HAnds (MUSHA) Hand II is a foldable 12-mm-diameter robotic gripper with 3 snake-like fingers, all equipped with force sensors for detection [46] However, the miniaturization and effective actuation of these 3-jaw grippers remain significant challenges, limiting their broader application in complex, minimally invasive surgeries. Hydraulic actuation has the potential to address these gaps by enabling a compact, high-force structure that maintains gripping performance even at reduced scales.

**Fig. 1.**
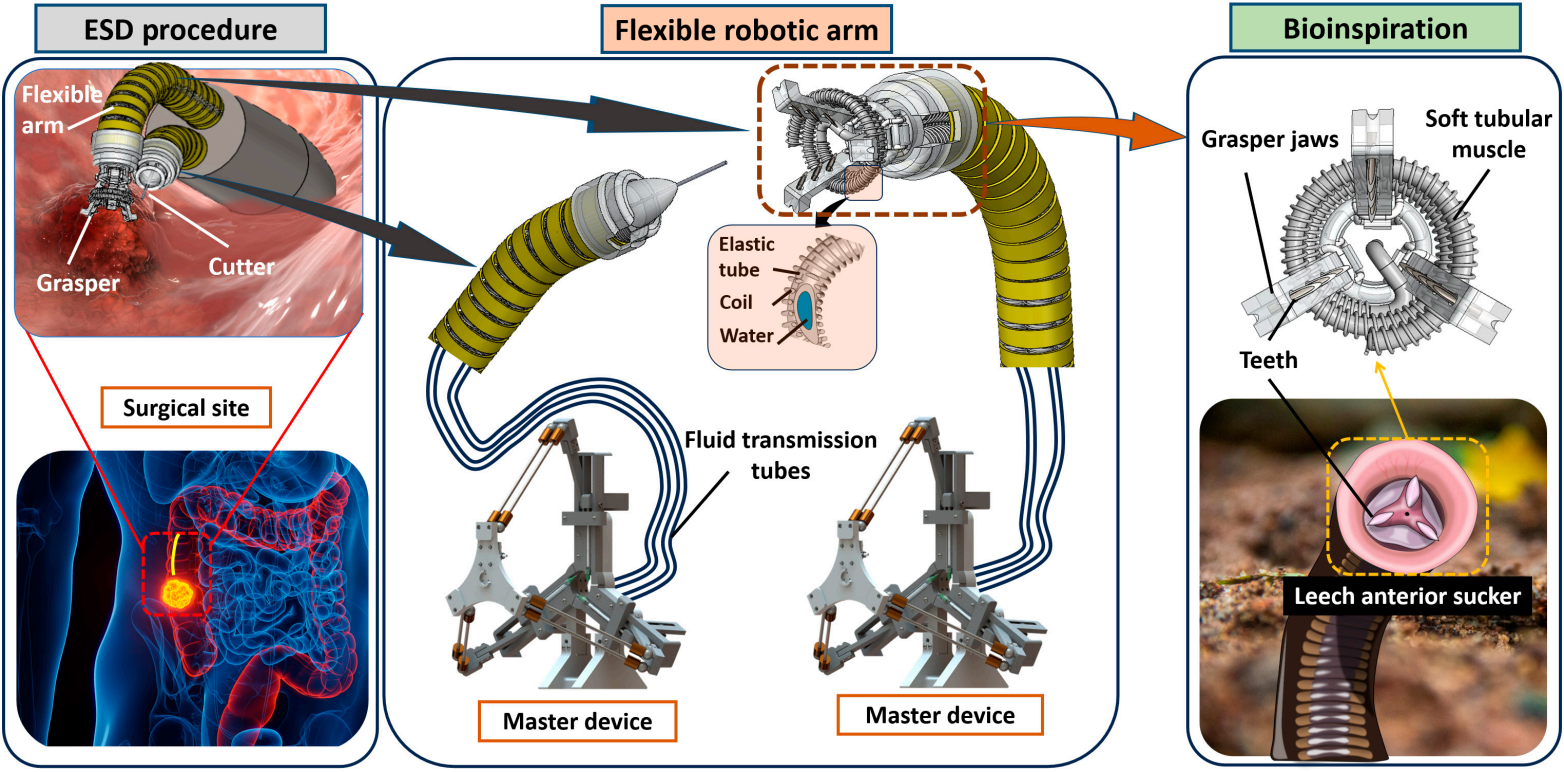
Overview of the soft endoscopic system featuring a hydraulic-driven 3 DOF-soft cutting arm (SCA) and a 3-jaw teleoperated soft grasper system (TSGS) for cutting and grasping lesions inside the colon, respectively. The soft manipulator (i.e., soft robotic arm) and grasper (i.e., end-effector) are operated by an electricity-free delta structure master device.

This paper introduces a new hydraulic-driven dual soft robotic system featuring a 3 DOF-soft cutting arm (SCA) and a 3-jaw teleoperated soft grasper system (TSGS). These components are purely operated by mechanical master–slave hydraulic architecture (Fig. 1) without the need for DC motors and microelectronic controllers. Instead of using cable-driven mechanisms associated with high friction and nonlinear hysteresis when working against torturous and complex paths, we employ miniature hydraulic soft actuators [47] to create omnidirectional manipulators. Thanks to its capability, the proximal soft robotic arms are capable of simultaneously inducing both bending and elongating movement without requiring external rotational and translational input from the proximal end. We also developed a new bioinspired 3-jaw grasper integrated with a soft manipulator tip for better gripping capability. This combination enables an ability to securely grip and manipulate tissues with even force distribution, thereby minimizing the risk of tissue damage compared to 2-jaw designs [45]. To drive the gripper, we employ a soft tubular muscle made from a micro-artificial muscle that expands and contracts radially to open and close the gripper jaws [48]. To manipulate the distal soft robotic arms, we developed a novel ergonomic master device featuring a delta structure that not only provides a large workspace, a high force ratio (output/input), and 4 DOFs but also enables precise one-to-one mapping with the movements of the slave soft robotic arms and actuation of the grasper jaws [49] without using any electronic components.

## Materials and Methods

### Design concept and working principle

The ESD procedure involves several steps: marking the lesion, followed by injecting fluid (Gelofusine) to elevate the mucosal layer, and then grasping, lifting, cutting, and removing the targeted tissue. In this work, we introduce a TSGS and an SCA, which are designed to perform removing lesion actions. Both instruments are connected to the master device via 1,600-mm-long transmission tubes (Fig. 1). The delta-structured master device remotely actuates the system using hydraulic driving, enabling precise ESD operations proximal to the colon. To enhance accessibility, the system’s surgical instruments incorporate soft hydraulic manipulators with a diameter of 5.5 mm and a compact 4-mm end-effector, which includes an electrocautery cutting knife and a grasper. This design facilitates efficient navigation through the colon, enabling effective lesion manipulation and removal [50]. Unlike conventional cable-driven systems, which suffer from force loss and increased control complexity at high bending angles due to friction, hydraulic actuators can transmit energy with negligible loss through pressure [22]. This ensures improved control stability and operational efficiency, particularly in deep intestinal regions. The system relies entirely on the delta-structured master device to transmit hydraulic force. It is designed with ergonomic features and force optimization, aiming to enhance ease of use for medical professionals and remove the necessity for DC motors or complex electronic controllers.

**Fig. 2.**
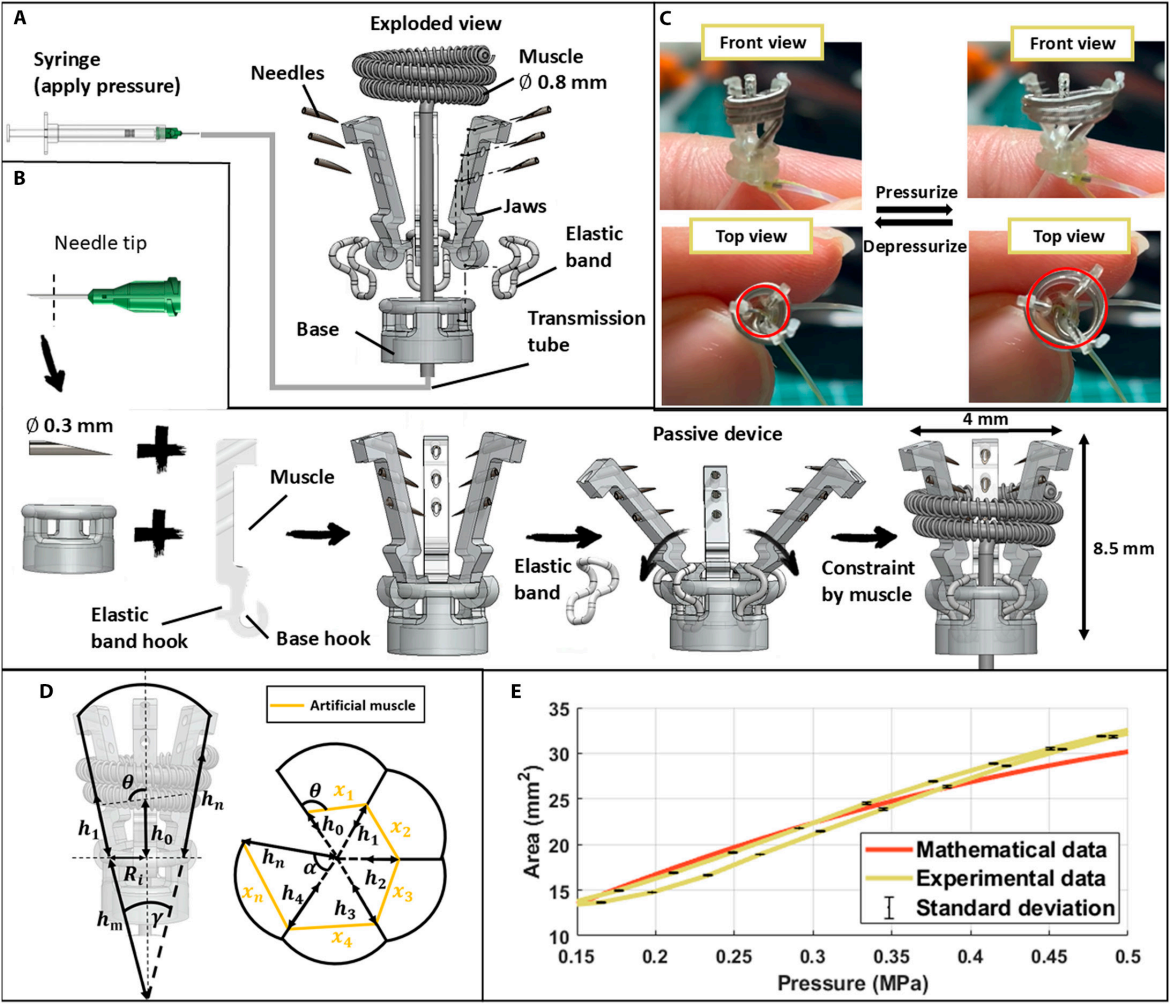
Design, analytical modeling, and characterization of the grasper. (A) Exploded view with detailed components. (B) Four main steps in the fabrication process. (C) Prototype and motion characterization with an image processing process. (D) Analytical model of the grasper’s motion with important parameters. (E) Comparison of experimental data and developed model for motion characterization.

To design a surgical gripper suitable for lesion manipulation, we drew bioinspiration from leeches, especially their mouth and teeth, which feed food using an anterior sucker consisting of 3 jaws arranged in a Y-shape at 120° intervals (Fig. 1) [51]. Each jaw has numerous small, sharp teeth that work together to create a precise, painless incision in the host’s skin [52]. Inspired by this unique occlusal mechanism from the leech, we develop the grasper with the 3 jaws evenly arranged around a circular shape similar to the leech’s anterior sucker, where its serrated teeth are arranged in a triangular formation (Fig. 2A). The teeth are designed to be tilted inward and hidden inside the jaw to prevent damage to surrounding tissues during the surgery. The detailed fabrication process of this grasper is shown in Fig. 2B and Note S1. To mimic the way a leech stretches and contracts its jaws during feeding, the movements of the grasper are triggered by a soft tubular hydraulic artificial muscle that is helically wrapped around the 3 jaws. This design provides a uniform compression force to close the grasper and allows the ability to grip the tumor effectively. For the opening, 3 pre-stretched elastic bands are attached to both the back of the jaws and the base, forming a passive structure that allows jaws to open automatically and evenly as the artificial muscle expands (Fig. 2C).

Since most tumors and mucosal elevations have rounded shapes [53]. Another advantage of the 3-jaw mechanism is its ability to enhance the grasper’s conformity to the rounded contours of these objects and evenly distribute the load across the lesion [42]. Compared with 2-jaw mechanisms, the 3-jaw design offers a self-centering function. This is due to the 3 jaws moving simultaneously toward the center, guiding the tissue into the center of the grasper and gripping evenly around its circumference. When the center of the grasper is maintained in a stable position, axial rotation does not affect the gripping performance. This eliminates the need for rotational adjustments within the manipulator to achieve an optimal grip, simplifying operation and improving stability during lesion manipulation. Additionally, the compact design of the grasper, with dimensions of 4 mm in diameter and 8.5 mm in length, further enhances its ability to navigate confined spaces [54].

To demonstrate the ability to perform a complete ESD procedure, we also developed the SCA, which features a soft robotic arm similar to that of the gripper and a cutting metal tip (0.3 mm in diameter) connected to an electrosurgical unit (SURTRON 120, Italy) as a monopolar electrode (Fig. 1). This flexible bending arm is used to steer the metal tip and approach the target tissue. The soft manipulator should possess both bending and axial elongation movements as a surgical instrument designed to operate in confined spaces (e.g., inside the colon). To achieve this, 3 hydraulic artificial muscles have been selected and arranged 120° apart, forming an omnidirectional manipulator with 3 DOFs where 2 DOFs are used for bending and 1 DOF is employed for translation. This configuration, along with its 35-mm length, allows the manipulator to effectively reach various positions within the colon with an approximate diameter of 40 mm [24,55]. To cut the tissue, the electrosurgical unit sent high-frequency sinusoidal electrical currents through the cable to generate heat at the tip of the cutter. When electrical energy contacts tissue, it causes rapid heating and vaporization of cells, leading to tissue cutting and ablation.

**Fig. 3.**
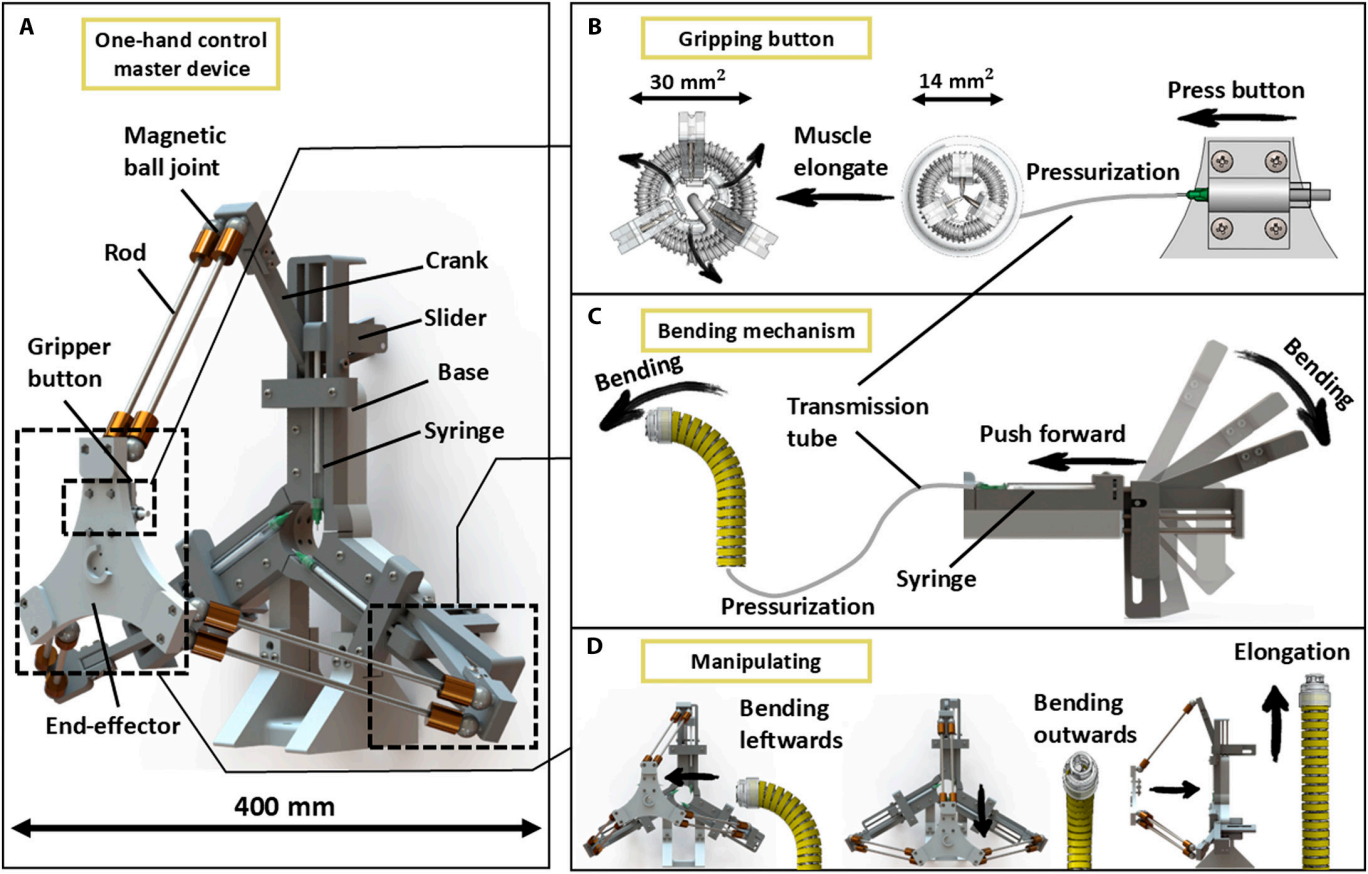
Working principle of the master device and grasper. (A) Overview of the master device and main components. (B) Gripping mechanism of the button operation. (C) Bending mechanism demonstrates the process of the master device operating the manipulator. (D) Manipulation process, showing the manipulator bending leftward, outward, and performing elongation under the master device operation.

To steer these soft robotic arms, we also developed an electricity-free master device, which features a delta construction including 3 syringes (i.e., the hydraulic pressure sources), as shown in Fig. 3A. The end-effector of the master device has 4 DOFs, 3 of which directly convert the input motions from the user to the slave grasper, and the last actuates the jaws of the grasper (Fig. 3B). To ensure smooth and precise control, the master device is articulated using magnetic ball joints. Combined with a parallelogram articulation structure, these ball joints allow for flexible translational movement of the end-effector. To apply the pressure needed for these precise movements, 3 Scotch Yoke mechanisms are integrated at the base of the master device. Each slider is connected to a syringe for pressurization by pushing the piston (Fig. 3C). This mechanism provides both a long-moment arm and a small force angle, resulting in a 1.45 force ratio to reduce the user’s effort [56] (Note S2). The movement of the end-effector drives all cranks of the Scotch Yoke mechanisms at the same time, enabling the end-effector to operate the soft manipulator (Fig. 3D) indirectly.

### Analytical model of the grasper

We developed an analytical model to examine the relationship between the muscle’s length and the opening area of the grasper with important parameters depicted in Fig. 2D. We assumed the grasper to have an initial shape as an inverted pyramid, When the pyramid is unfolded and laid flat on a plane, it becomes fan-shaped with equal sides. The length of the muscle is separated into *n* segments, where each segment $x_{i}$ represents the distance between 2 consecutive jaws. Therefore, the total muscle length $\;L_{m}$, is defined as the sum of all segments: $L_{m}=\sum_{i=1}^{n}x_{i}.$ The distance between the intersection points of 2 jaws and the intersection points of the base and jaws $h_{m}$, varies depending on the opening angle of the grasper γ and the base radius $R_{i}$, designed to be 2 mm in this model.

$$h_{m}=\frac{2\pi R_{i}}{3}\text{sin}\left(\gamma /2\right)$$ (1)

The distance between the connection of the base and the muscle segment $h_{i}≔\left[h_{m},\;\varphi ,\;\gamma \right]\in {\mathbb{R}}^{3}$ is calculated by trigonometric functions. Here,$\;h_{n}\;$represents the distance between the intersection points of the base and jaws and the intersection points of the muscle and jaws. The variable θ is the wrapping angle of the muscle, which is 83° in this design. Additionally, $h_{0}$ is defined as the distance between the intersection points of the base and jaws and the starting point of the muscle, set at 2 mm.

$$h_{i}\left(h_{m}\right)=\frac{2\left(h_{i-1}+h_{m}\right)\text{sin}\left(\frac{\gamma }{2}\right)\text{sin}\left(90-\theta +\frac{\gamma }{2}\right)}{\text{sin}\left(\theta -\gamma \right)}+h_{i-1}$$ (2)

Given an initial muscle length of 30 mm, the opening angle of the jaws can be determined in relation to the actual muscle length as follows:

$$\begin{array}{c} L_{m}=\sum_{i=1}^{n}x_{i}=\\ \sum_{i=1}^{n}2\left(h_{i-1}+h_{m}\right)\text{sin}\left(\frac{\gamma }{2}\right)\text{cos}\left(90-\theta +\frac{\gamma }{2}\right)+\left(h_{i}-h_{i-1}\right)\text{cos}\left(\theta -\gamma \right) \end{array}$$ (3)

The opening area A, which is a key parameter for understanding the grasper’s functionality, is calculated by:

$$A=\pi {\left(3\mathrm{h\gamma}/\left(2\pi \right)\right)}^{2}$$ (4)

### Experimental validation

#### Characterization of the grasper motion

The grasper’s movements were captured from top views using a camera (NIKON D7500, Japan), facilitating a detailed visual analysis (Movie S1). Gripping motions of the grasper were activated by a linear slider (Zaber, Canada), which precisely controlled the input pressure with a sinusoidal motion with a peak-to-peak amplitude of 0.58 MPa and a frequency of 0.5 Hz. As the applied pressure varies, the grasper initiates its opening and closing actions. The grasper’s workspace was delineated using image analysis techniques. For enhanced visual distinction from the background and the grasper itself during analysis, the tips of the grasper jaws were painted red. This color marking allowed for accurate segmentation and analysis within MATLAB, where RGB masking techniques were employed to isolate and examine the 3 red regions. A circle could be delineated representing the grasper’s opening area by constructing centroids of these identified areas. Fig. S3A illustrates the methodology of image analysis and how the opening area varies in response to changes in the input pressure.

To characterize the analytical model of the opening area, an experiment was conducted to analyze the relationship between pressure and elongation for a muscle with a diameter of 0.8 mm. A 30-mm length muscle was securely fixed on a workbench and connected to a monofilament (Fig. S4). A pressure sensor was installed between the syringe and the muscle to measure the applied pressure. The monofilament was attached to the rotating pin of an encoder (US Digital, USA) to detect its displacement. The opposite end of the monofilament was secured with a fixed screw. To ensure the monofilament remained taut throughout the experiment, a spring with an outside diameter of 0.5 mm was used to replace a segment of the monofilament, providing a consistent compression force as the muscle elongated and the spring shortened.

#### Characterization of the working space of the manipulator

The manipulator was positioned 20 cm from a wide-range magnetic transmitter and securely locked in a 3-dimensional (3D)-printed part with an 8-mm hollow channel to ensure precise position data. To mitigate electromagnetic (EM) interference, nearby electronic devices, power lines, and metal objects were removed, and the sensor was placed 10 mm above a helical coil (Fig. S3B). The system utilized 3 BD Luer-Lok 1-ml syringes (inner diameter of 4.5 mm), each driven by a DC micromotor (model 3272G024CR with encoder IE3-1024, Faulhaber, Germany) and ball screw mechanism (MISUMI, Japan), all controlled by MATLAB Simulink. Pressure sensors (40PC Series, Honeywell, USA) were connected to each muscle to monitor the relationship between pressure and manipulator movement. Each muscle represents one pressure (P1, P2, and P3). To collect the range of radial working space, a sinusoidal pressure function (0.2, 0.4, and 0.6 MPa) with varying phases (2/3π and 4/3π) was applied to each muscle to achieve rotation.

#### Characterization of the contraction force of the manipulator

A calibrated miniature load cell (FUTEK, USA) detected the contraction force. Data collection and analysis were facilitated using a QPIDe data acquisition device (QUANSER, Canada) integrated with MATLAB Simulink. The manipulator was secured using a bench vice, and the load cell was fixed onto a 3D-printed base. A monofilament line connected the manipulator to the load cell (Fig. S5A). Three syringes were mounted onto a motorized linear slider (Zaber, Canada) using a 3D-printed component. This configuration allowed the linear slider to simultaneously push or withdraw all syringes. The linear slider was gradually controlled to apply hydraulic pressure to the manipulator, elongating it until a pressure of 0.6 MPa was achieved, after which the hydraulic pressure was released to generate a contraction force, a process that was repeated 8 times.

#### Characterization of the steering of the master–slave device

The master–slave system was set up by securing the master device onto an optical table, while the manipulator was positioned 15 cm in front of an N920 camera (Nano Shield) for precise visual tracking (Fig. S5B). The camera was connected to a monitor to observe and film the manipulator’s motion. The operator manipulated the master device while observing the manipulator’s position on the screen, aiming to align it with the predefined patterns as closely as possible. Predefined patterns, including a rectangle and a circle, were displayed translucently on the monitor to guide the manipulator’s movements. Each pattern was repeated 3 times, and any deviations were recorded for further analysis.

## Results

### Characterization

#### Opening area of the grasper

An experiment was conducted using the setup shown in Fig. S3A to characterize the operational workspace of the surgical grasper. The grasper’s movements were captured from a top-down view using a digital single-lens reflex (DSLR) camera (NIKON D7500, Japan). At the same time, a linear slider (Zaber, Canada) controlled the sinusoidal input pressure to drive the motion of grasper jaws. The tips of the grasper were painted red to enhance accuracy during workspace analysis using RGB masking in MATLAB. The results showed that the grasper’s opening area increased from an initial 12.5 to 31.2 mm^2^ at a pressure of 0.5 MPa, representing an expansion factor of 2.5 times (Fig. 2E). The increase in opening area was observed starting at 0.15 MPa, which can be due to residual air bubbles in the transmission or silicone tube. The transmission of pressure within the tube is delayed by bubbles, resulting in the failure of pressure to be transmitted to the artificial muscle in time. As pressure increases and the air bubbles are gradually compressed, the system stabilizes, allowing for more consistent expansion and control of the grasper’s opening area.

To validate the mathematical model, an experiment was conducted to analyze the relationship between pressure and elongation for a muscle with a diameter of 0.8 mm. The Zaber applied a sinusoidal displacement to the syringe piston during the experiment. A data acquisition device collected the displacement and pressure data simultaneously. Fig. S4 shows the experimental data and setup. The relationship equation is simplified to a quadratic equation, which is shown below:

$$y=-40.38x^{2}+63.8x-0.77$$ (5)

where *y* represents the displacement of the muscle in millimeters, *x* represents the pressure in megapascals, and the coefficient of regression analysis *R*^2^ is 0.998.

These results indicate that the model used to analyze the relationship between pressure and displacement is highly accurate. Based on the above relationship, the mathematical model for the grasper’s opening area as a function of muscle length was employed to derive the relationship between muscle inner pressure and the opening area of the grasper. The equations are solved by the symbolic math toolbox of MATLAB, yielding a quadratic curve. The derived mathematical data were compared with the experimental data, as illustrated in Fig. 2E. *R*^2^ is 0.947, indicating a strong correlation and supporting the model’s accuracy in predicting the grasper’s opening area under varying pressure conditions.

#### Working space of the manipulator

**Fig. 4.**
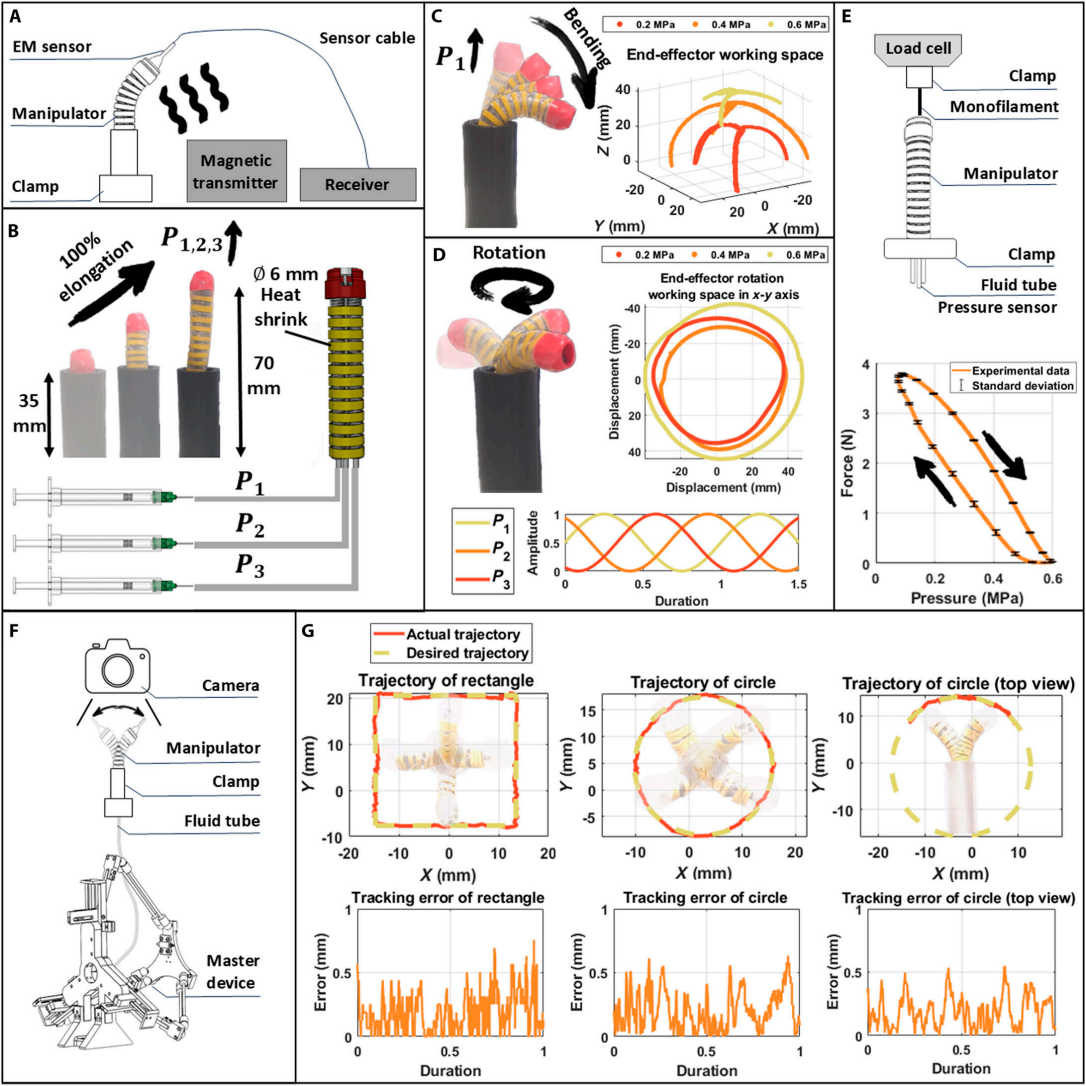
Design, working principle, and characterization of the manipulator. (A) Experiment setup for detecting the working space of the manipulator. (B) Elongation motion and working principle of the manipulator. (C) Bending motion and working space of the manipulator. (D) Rotation motion and working space of the manipulator. (E) Experiment setup and results of the contraction force of the manipulator. (F) Experiment setup for steering. (G) Actual and desired trajectory results and errors during one cycle.

An EM tracking system (Trak STAR, TRACKLAB – FREEDSPACE Ltd, Canada) was employed to track the end-effector positions to fully characterize the operational workspace of the manipulator. An EM 6-DOF sensor (model 90) was attached to the manipulator’s end-effector to transmit its 3D coordinates in real time during the experimental process (Fig. 4A). From Fig. 4B, the manipulator elongated from the chamber when all 3 muscles were pressurized simultaneously. Initial pressures of 0.2, 0.4, and 0.6 MPa were respectively applied, followed by adjustments to the inner pressures of each muscle to make the manipulator bend (Fig. 4C) and rotate (Fig. 4D). The results demonstrated that the actuator could elongate to 70 mm (100% elongation) and achieve a 40-mm-diameter rotating trajectory under initial pressures of 0.2, 0.4, and 0.6 MPa, indicating that the working space of the manipulator is sufficient for ESD surgeries.

#### Contraction force of the manipulator

The contraction force experiment was conducted to evaluate the capability of a manipulator to lift tissue, as depicted in Fig. 4E. Compared to non-extensible graspers, our grasper utilizes contraction forces from elongation rather than relying on bending forces to lift the tissue. The experiment setup involved using a load cell (FUTEK, USA) to measure contraction force, where a motorized linear slider (Zaber, Canada) was applied, and hydraulic pressure was withdrawn to the manipulator. The force generated during pressure withdrawal and the decrease in force during pressure increase were recorded, with the force–pressure curve monitored for nonlinear hysteresis. The experiment demonstrated that the manipulator could generate a maximum contraction force of 3.88 N at a hydraulic pressure drop of 0.52 MPa, above the 2.26 N threshold required for effective tissue manipulation in ESD procedures [57]. The consistency across multiple trials also indicates the reliability of the system in generating predictable forces under controlled conditions. The nonlinear hysteresis observed is primarily attributed to the viscoelastic nature of the elastomer in the artificial muscles, which exhibits both viscosity and elasticity. Unlike elastic deformation, the energy consumed to overcome viscosity cannot be recovered [58]. Therefore, released pressure generates less force compared to applied pressure.

#### Steering of master–slave device

A series of path-following tests were conducted to evaluate the system’s steering accuracy using predefined patterns in both front and top views (Fig. 4F). The operator manipulated the master device while monitoring the manipulator’s performance on the screen and following the trajectory of redefined patterns as closely as possible. Any deviations from the intended trajectory were recorded for later analysis. The accuracy of the system was assessed by performing 3 different path-following exercises: 2 in the front view and 1 in the top view (Fig. 4G). The recorded video footage was analyzed to measure the tracking errors. For the rectangular path, the system achieved a mean tracking error of 0.1889 mm, demonstrating the ability of the manipulator to maintain precise alignment with straight lines and sharp corners. The circular path resulted in mean tracking errors of 0.2083 mm in the front view and 0.1899 mm in the top view, indicating a slightly greater difficulty in maintaining consistent motion along curved paths. Nonetheless, these minimal errors reflect the high accuracy and performance that the TSGS offers. These results validate the effectiveness of the master–slave operation system in following predefined paths with minimal deviations, proving the precision of the manipulator in real surgical applications. The consistently low tracking errors demonstrate the system’s ability to accurately manage complex movements, ensuring safe and effective manipulation of tissues during ESD procedures.

#### Response time and durability

In addition to the core performance evaluation, we conducted 2 complementary tests to assess the response time and durability of the TSGS. First, the response time experiment evaluated the delay between force application on the master device and force detection on the slave instrument. Across 11 trials under the same conditions, the average response time was 0.0895 s with a standard deviation of 0.0227 s (Note S5). The response time is lower than the speculated maximum safe latency period of around 150 ms, indicating rapid energy transfer and potentially promoting safer operation [59]. Second, the durability experiment evaluated the system’s ability to be repeatedly actuated during prolonged surgical procedures. The soft manipulator arm was subjected to continuous commands to reach the designated target at nearly 0.2 Hz for more than 40 min (Note S6), which is more than twice the duration of the average circumferential cutting phase [60]. The displacement offset in the *y*-axis was only 0.9%. This finding indicates that the TSGS has the potential to maintain stable performance and integrity over multiple actuation cycles, which is critical for longer procedures and potential reusability in clinical settings.

### Demonstration

#### In vitro experiment

**Fig. 5.**
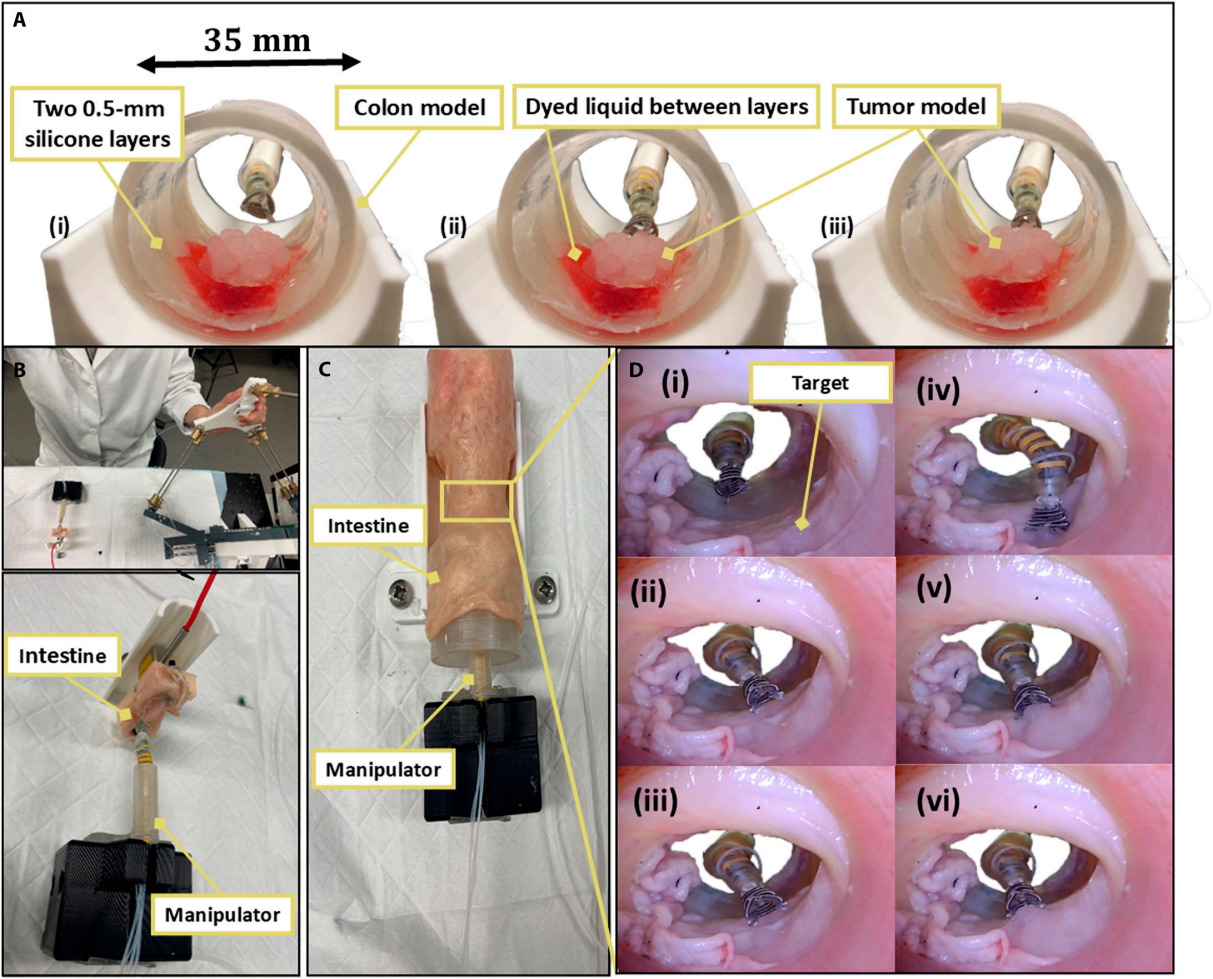
In vitro and ex vivo experiments demonstrate grasper operation and tissue manipulation. (A) In vitro experiment of operating the grasper to grip the tumor model by the master device. (B) Lifting porcine large intestine. (C) An ex vivo experiment was set up to grasp the target inside a large porcine intestine*.* (D) Ex vivo experiment grasping procedure.

We conducted an in vitro experiment to evaluate the steering capabilities of the manipulator and the gripping efficiency of the grasper mechanism (Fig. 5A). The colon model used for the experiment consists of 2 silicone layers, each 0.5 mm thick, representing the mucosal and submucosal layers. These layers were fabricated using EcoFlex 00-10 (Smooth-On, Inc, USA), chosen for its Young’s modulus, which closely approximates that of the colon’s mucosal layer. A laterally spreading tumor model, fabricated from Dragon Skin 10 (Smooth-On, Inc, USA) with a 20-mm diameter and a 6.8-mm height, was adhered to the mucosal layer using Sil-Poxy adhesive (Smooth-On, Inc, USA). The silicone layers were then affixed to a 3D-printed model with a 35-mm inner diameter. A dyed liquid was injected between them to simulate the separation between the mucosal and submucosal layers.

The manipulator was inserted into the colon model, and the master device, operated with a single hand, was used to steer the grasper toward the tumor model (Movie S3). Upon approaching the target, the grasper’s jaws were opened using the master device’s trigger. The end-effector of the master device was then advanced, allowing the grasper to envelop part of the lesion. After reaching the target, the pressure was released, enabling the jaws to grip the tumor securely. By retracting the end-effector, the grasper lifted both the tumor and the attached mucosal layer, causing the dyed liquid to converge toward the center. Finally, the trigger was pressed again to release the tumor model.

#### Ex vivo experiment

Following the in vitro experiment, a series of ex vivo experiments were conducted using a large porcine intestine purchased from a local supermarket (Coles, Randwick, Sydney, NSW 2052, Australia) to further evaluate the system’s performance. The first ex vivo experiment focused on assessing the ability to apply a contraction force to the real intestine (Fig. 5B). The second experiment aimed to evaluate the system’s capability to achieve both grasping functionality and telemanipulation within the intestine (Fig. 5C). Two 3D-printed models with a 40-mm outside diameter were inserted inside the intestine to extend the working space as a stabilization mechanism. A small incision was made in the submucosal layer to insert a small piece of silicone between the submucosal layer and the muscle layer, simulating a target. A camera (BlueFire, China) was positioned to capture the grasping procedure from the opposite side of the grasper (Movie S4). The intestine, camera, and grasper were all securely fixed to an optical platform. Fig. 5D illustrates the sequential steps of the grasping process, from steering to grasping to lifting, replicating the procedures performed during the in vitro experiment.

#### Ex vivo experiment with electrosurgical cutting

**Fig. 6.**
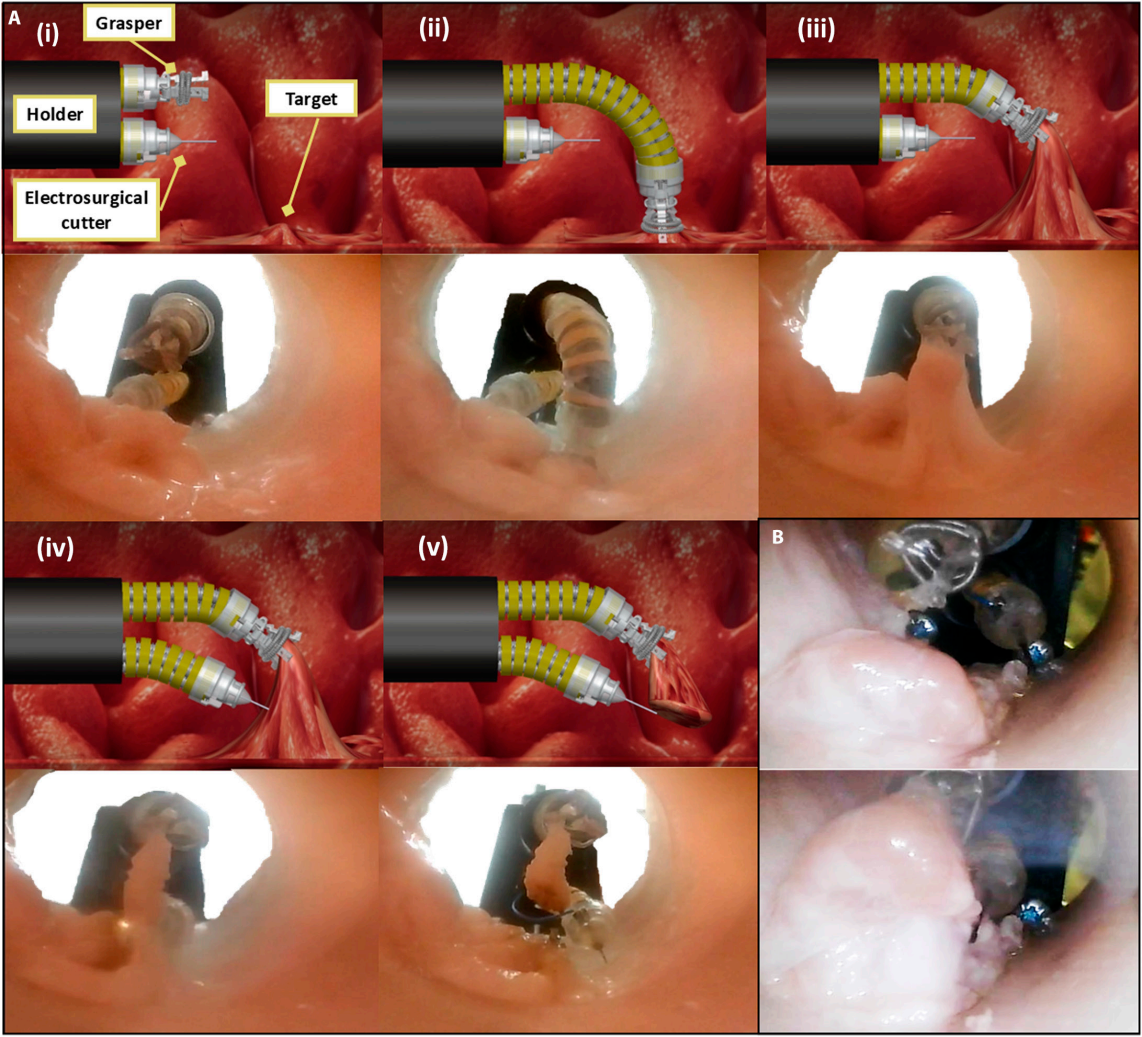
Ex vivo experiment of the grasper and the electrosurgical cutter. (A) Set up and demonstrate the grasper and cutter positioned in a dual-channel holder for performing ESD on a large porcine intestine. The top row (i to iii) shows the gripping and lifting of the target tissue, while the bottom row (iv and v) illustrates the electrosurgical cutting process and tissue removal. (B) Demonstration of the grasper securely gripping a large tissue sample during the ESD procedure.

In this ex vivo experiment, targeted tissue was successfully removed from a porcine large intestine, as outlined in the procedural workflow depicted in Fig. 6A. Both the grasper and the cutter were mounted in a custom holder with 2 hollow channels, positioning the grasper above the cutter (Movie S5). The top 3 images illustrate the initial stages of tissue manipulation, where the grasper precisely grips and elevates the target tissue to provide clear access for the cutter. This step mirrors the standard submucosal injection and lesion elevation phase in ESD, enabling the separation of the lesion from the underlying muscular layer. The bottom 2 images in Fig. 6A depict the subsequent electrosurgical cutting process, where the cutter excises the tissue while simultaneously sealing the surrounding area, completing the lesion removal. In addition to gripping small tissue, Fig. 6B evaluated the grasper’s ability to manipulate large-scale tissue while working in tandem with the cutter.

## Discussion

This paper presents a soft hydraulic drive system, TSGS, for ESD. Teleoperation is achieved by the pressure transmission principle, which overcomes the limitations of conventional cable-driven systems, such as nonlinear friction, hysteresis, and bulkiness. In addition, this system designs a 3-jaw grasper inspired by the front sucker of leeches to achieve uniform force distribution and eliminate rotational adjustment, as well as a Delta structure master device that provides precise, nonelectrical control. Experimental results demonstrated the system’s ability to generate a maximum contraction force of 3.88 N under a pressure drop of 0.52 MPa, exceeding the 2.26 N threshold required for effective tissue manipulation in ESD. Path-following tests indicate that the system can grasp and cauterize in the colon with submillimeter accuracy (<~0.2 mm)

The TSGS was compared with existing surgical systems across critical metrics, as summarized in Table 1. The TSGS achieves a higher lifting force of 3.88 N, which exceeds systems like FPCW (1.54 N) [61], STRAS (1.5 N) [20], and Lau et al. [62]. This enhanced force capability enables TSGS to effectively manipulate larger lesions. At the same time, the average trajectory error of TSGS was 0.2 mm, and the response time was 0.1 s, which can meet the surgery requirements. Although the accuracy is slightly greater than RMIS (0.15 mm), the system maintains a compact diameter of 5.5 mm, enabling access to narrow colon segments [63]. Unlike systems limited to the transverse colon or descending colon due to rigidity or friction loss, TSGS can operate at any location in the colon, including the cecum and ileocecal valve. Clinically, the ability to perform endoscopic surgery in the cecum is crucial for minimally invasive treatment of proximal lesions, which account for 42.4% of CRCs [64]. Ex vivo experiments demonstrated that TSGS and SCA can perform tissue grabbing, lifting, and cutting functions in the large intestine. In addition, in vitro experiments demonstrated that TSGS can work effectively in the limited space of the colorectal region.

**Table 1.** Robot-assisted dual-instrument surgical system comparisons

	Lifting force (N)	Average trajectory error (mm)	Diameter (mm)	Reach (%)
FPCW [61]	1.54	0.52	3.0	33
RMIS [63]	10	0.15	9.5	75
STRAS [20]	0.9	3.9	4.3	34
K-FLEX [65]	3		3.7	62.5
Lau et al. [62]	0.47		~6	62.5
TSGS (this work)	3.88	0.2	5.5	100

Future work will focus on integrating haptic feedback during tissue manipulation, providing the operator with better control during delicate procedures. Furthermore, integrating advanced image processing technologies into TSGS can enhance visual feedback and improve stabilization mechanisms, minimizing the impact of small, involuntary movements caused by respiration or muscle twitches. With its compact and compliant design, the TSGS offers the potential for adaptation in other NOTES procedures, extending its applicability beyond ESD surgery to broader surgical contexts. In order to expand the application of TSGS to other surgeries, such as the transnasal endoscopic surgery and transurethral resection of bladder tumor mentioned above, the surgical instruments of TSGS need to be further reduced and shortened to accommodate the size of these natural cavities. Second, specialized or interchangeable end-effectors may be required to handle diverse tissue types and perform surgical tasks, such as suction or suturing.

## Data Availability

The data are available upon reasonable request.

## References

[1] Ferlay J, Soerjomataram I, Dikshit R, Eser S, Mathers C, Rebelo M, Parkin DM, Forman D, Bray F. Cancer incidence and mortality worldwide: Sources, methods and major patterns in GLOBOCAN 2012. Int J Cancer. 2015;136(5):E359–E386. 25220842 10.1002/ijc.29210

[2] Alkatout I, Mechler U, Mettler L, Pape J, Maass N, Biebl M, Gitas G, Laganà AS, Freytag D. The development of laparoscopy—A historical overview. Front Surg. 2021;8:799442. 34977146 10.3389/fsurg.2021.799442 PMC8714650

[3] Cianchetti M, Laschi C, Menciassi A, Dario P. Biomedical applications of soft robotics (in English). Nat Rev Mater. 2018;3(6):143–153.

[4] Maeso S, Reza M, Mayol JA, Blasco JA, Guerra M, Andradas E, Plana MN. Efficacy of the Da Vinci surgical system in abdominal surgery compared with that of laparoscopy: A systematic review and meta-analysis (in English). Ann Surg. 2010;252(2):254–262. 20622659 10.1097/SLA.0b013e3181e6239e

[5] Vitiello V, Lee SL, Cundy TP, Yang GZ. Emerging robotic platforms for minimally invasive surgery. IEEE Rev Biomed Eng. 2013;6:111–126. 23288354 10.1109/RBME.2012.2236311

[6] Fuchs K-H, Breithaupt W. Transgastric small bowel resection with the new multitasking platform EndoSAMURAI™ for natural orifice transluminal endoscopic surgery. Surg Endosc. 2012;26:2281–2287. 22395953 10.1007/s00464-012-2173-z

[7] Berthet-Rayne P, Gras G, Leibrandt K, Wisanuvej P, Schmitz A, Seneci CA, Yang GZ. The i^2^snake robotic platform for endoscopic surgery. Ann Biomed Eng. 2018;46(10):1663–1675. 29948372 10.1007/s10439-018-2066-y PMC6154016

[8] Ji R, Yang JL, Yang XX, Fu SC, Li LX, Li YQ, Zuo XL. Simplified robot-assisted endoscopic submucosal dissection for esophageal and gastric lesions: A randomized controlled porcine study (with videos). Gastrointest Endosc. 2022;96(1):140–147. 35065045 10.1016/j.gie.2022.01.004

[9] Hwang M, Lee SW, Park KC, Sul HJ, Kwon DS. Evaluation of a robotic arm-assisted endoscope to facilitate endoscopic submucosal dissection (with video). Gastrointest Endosc. 2020;91(3):699–706. 31751551 10.1016/j.gie.2019.11.014

[10] Ngu JC, Tsang CB, Koh DC. The da Vinci Xi: A review of its capabilities, versatility, and potential role in robotic colorectal surgery. Robot Surg. 2017;4:77–85. 30697566 10.2147/RSRR.S119317 PMC6193435

[11] Kwok KW, Wurdemann H, Arezzo A, Menciassi A, Althoefer K. Soft robot-assisted minimally invasive surgery and interventions: Advances and outlook (in English). Proc IEEE. 2022;110(7):871–892.

[12] Ullah S, Ali FS, Liu BR. Advancing flexible endoscopy to natural orifice transluminal endoscopic surgery. Curr Opin Gastroenterol. 2021;37(5):470–477. 34091533 10.1097/MOG.0000000000000753

[13] Rao GV, Reddy DN, Banerjee R. NOTES: Human experience. Gastrointest Endosc Clin N Am. 2008;18(2):361–370. 18381176 10.1016/j.giec.2008.01.007

[14] Baekelandt J, de Mulder PA, le Roy I, Mathieu C, Laenen A, Enzlin P, Weyers S, Mol BW, Bosteels JJ, Postoperative outcomes and quality of life following hysterectomy by natural orifice transluminal endoscopic surgery (NOTES) compared to laparoscopy in women with a non-prolapsed uterus and benign gynaecological disease: A systematic review and meta-analysis, Eur J Obstet Gynecol Reprod Biol*.* 2017;208:6–15. 27880893 10.1016/j.ejogrb.2016.10.044

[15] Karagul S, Tardu A. Who is suitable for natural orifice specimen extraction (NOSE) following laparoscopic colorectal surgery: A narrative review. Ann Laparosc Endosc Surg. 2022;7: 10.21037/ales-22-17.

[16] Draganov PV, Wang AY, Othman MO, Fukami N. AGA Institute clinical practice update: Endoscopic submucosal dissection in the united states. Clin Gastroenterol Hepatol. 2019;17(1):16–25 e1. 30077787 10.1016/j.cgh.2018.07.041

[17] Zorn L, Nageotte F, Zanne P, Legner A, Dallemagne B, Marescaux J, de Mathelin M. A novel telemanipulated robotic assistant for surgical endoscopy: Preclinical application to ESD, IEEE Trans Biomed Eng*,* 2018;65(4):797–808. 28678698 10.1109/TBME.2017.2720739

[18] Shang J, Leibrandt K, Giataganas P, Vitiello V, Seneci CA, Wisanuvej P, Liu J, Gras G, Clark J, Darzi A, et al. A single-port robotic system for transanal microsurgery—Design and validation. IEEE Robot Autom Lett. 2017;2(3):1510–1517.

[19] Zorn L, Nageotte F, Zanne P, Legner A, Dallemagne B, Marescaux J, de Mathelin M. A novel telemanipulated robotic assistant for surgical endoscopy: Preclinical application to ESD. IEEE Trans Biomed Eng. 2017;65(4):797–808. 28678698 10.1109/TBME.2017.2720739

[20] De Donno A, Zorn L, Zanne P, Nageotte F, de Mathelin M. Introducing STRAS: A new flexible robotic system for minimally invasive surgery. In: *2013 IEEE International Conference on Robotics and Automation*. Karlsruhe (Germany): IEEE; 2013. p. 1213–1220.

[21] Li Z, Wang L, Wu L, Alambeigi F, Cheng SS. Flexible surgical robotics: Design, modeling, sensing and control. Front Robot AI. 2022;9:854024. 35516787 10.3389/frobt.2022.854024 PMC9062025

[22] Hassani V, Tjahjowidodo T, Do TN. A survey on hysteresis modeling, identification and control (in English). Mech Syst Signal Process. 2014;49(1-2):209–233.

[23] Le HM, Do TN, Phee SJ. A survey on actuators-driven surgical robots. Sens Actuators A Phys. 2016;247:323–354.

[24] Nguyen CC, Hoang TT, Davies J, Phan PT, Thai MT, Nicotra E, Abed AA, Tran HA, Truong TA, Sharma B, et al. Soft fibrous syringe architecture for electricity-free and motorless control of flexible robotic systems. Adv Sci. 2024;11(39):2405610. 10.1002/advs.202405610 PMC11497036 39159303

[25] Cianchetti M, Ranzani T, Gerboni G, De Falco I, Laschi C, Menciassi A. STIFF-FLOP surgical manipulator: Mechanical design and experimental characterization of the single module. In: *2013 IEEE/RSJ international conference on intelligent robots and systems*. Karlsruhe (Germany): IEEE; 2013. p. 3576–3581.

[26] Nguyen CC, Thai MT, Hoang TT, Davies J, Phan PT, Zhu K, Wu L, Brodie MA, Tsai D, Ha QP, et al. Development of a soft robotic catheter for vascular intervention surgery. Sens Actuators A Phys. 2023;357: Article 114380.

[27] Dreyfus R, Boehler Q, Lyttle S, Gruber P, Lussi J, Chautems C, Gervasoni S, Berberat J, Seibold D, Ochsenbein-Kölble N, et al. Dexterous helical magnetic robot for improved endovascular access. Sci Robot. 2024;9(87):eadh0298. 38354258 10.1126/scirobotics.adh0298

[28] Zhou C, Yang Y, Wang J, Wu Q, Gu Z, Zhou Y, Liu X, Yang Y, Tang H, Ling Q, et al. Ferromagnetic soft catheter robots for minimally invasive bioprinting. Nat Commun. 2021;12(1):5072. 34417473 10.1038/s41467-021-25386-w PMC8379157

[29] Luo X, Song D, Zhang Z, Wang S, Shi C. A novel distal hybrid pneumatic/cable-driven continuum joint with variable stiffness capacity for flexible gastrointestinal endoscopy. Adv Intell Syst. 2023;5(6):2200403.

[30] Yang ZX, Li Z. Magnetic actuation systems for miniature robots: A review. Adv Intell Syst. 2020;2(9):2000082.

[31] Yu N, Hollnagel C, Blickenstorfer A, Kollias SS, Rierner R. Comparison of MRI-compatible mechatronic systems with hydrodynamic and pneumatic actuation. IEEE/ASME Trans Mechatron. 2008;13(3):268–277.

[32] Focchi M, Guglielmino E, Semini C, Parmigianni A, Tsagarakis N, Vanderborght. Water/air performance analysis of a fluidic muscle. In: *2010 IEEE/RSJ international conference on intelligent robots and systems*. Taipei (Taiwan): IEEE; 2010. p. 2194–2199.

[33] Meller MA, Bryant MJ, Garcia E. Energetic and dynamic effects of operating fluid on fluidic artificial muscle actuators. In: *Smart materials, adaptive structures and intelligent systems*. Snowbird (UT): American Society of Mechanical Engineers; 2013. Vol. 56048, p. V002T06A019.

[34] Xavier MS, Tawk CD, Zolfagharian A, Pinskier J, Howard D, Young T, Lai J, Harrison SM, Yong YK, Bodaghi M, et al. Soft pneumatic actuators: A review of design, fabrication, modeling, sensing, control and applications. IEEE Access. 2022;10:59442–59485.

[35] Bruns TL, Remirez AA, Emerson MA, Lathrop RA, Mahoney AW, Gilbert HB, Liu CL, Russell PT, Labadie RF, Weaver KD, et al. A modular, multi-arm concentric tube robot system with application to transnasal surgery for orbital tumors. Int J Robot Res. 2021;40(2-3):521–533.

[36] Cao Y, Shi Y, Hong W, Dai P, Sun X, Yu H, Xie L. Continuum robots for endoscopic sinus surgery: Recent advances, challenges, and prospects. Int J Med Robot. 2023;19(2): Article e2471. 36251333 10.1002/rcs.2471

[37] Kuang H, Wang X, Zhao C, Zhu C, Xu K. Proof-of-concept development of the distal module of a cystoscope transurethral continuum surgical robotic system. 2024;9(11):10002–10009.

[38] Hu X, Chen A, Luo Y, Zhang C, Zhang E. Steerable catheters for minimally invasive surgery: A review and future directions. Comput Assist Surg. 2018;23(1):21–41. 10.1080/24699322.2018.1526972 30497292

[39] Chiu PWY, Ho KY, Phee SJ. Colonic endoscopic submucosal dissection using a novel robotic system (with video). Gastrointest Endosc. 2021;93(5):1172–1177. 32991869 10.1016/j.gie.2020.09.042

[40] Thompson CC, Ryou M, Soper NJ, Hungess ES, Rothstein RI, Swanstrom LL. Evaluation of a manually driven, multitasking platform for complex endoluminal and natural orifice transluminal endoscopic surgery applications (with video). Gastrointest Endosc. 2009;70(1):121–125. 19394008 10.1016/j.gie.2008.11.007

[41] Schuler PJ, Hoffmann TK, Veit JA, Rotter N, Friedrich DT, Greve J, Scheithauer MO. Hybrid procedure for total laryngectomy with a flexible robot-assisted surgical system. Int J Med Robot. 2017;13(2): Article e1749. 10.1002/rcs.1749 27196407

[42] Hernandez J, Sunny MSH, Sanjuan J, Rulik I, Zarif MII, Ahamed SI, Ahmed HU, Rahman MH. Current designs of robotic arm grippers: A comprehensive systematic review. Robotics. 2023;12(1):5.

[43] Samadikhoshkho Z, Zareinia K, Janabi-Sharifi F. A brief review on robotic grippers classifications. In: 2019 IEEE Canadian Conference of Electrical and Computer Engineering (CCECE). Edmonton (Canada): IEEE; 2019. p. 1–4.

[44] Mirbagheri A, Farahmand F. Design, analysis, and experimental evaluation of a novel three-fingered endoscopic large-organ grasper. J Med Devices. 2013;7(2): Article 025001.

[45] Oshima R, Takayama T, Omata T, Kojima K, Takase K, Tanaka N. Assemblable three-fingered nine-degrees-of-freedom hand for laparoscopic surgery. IEEE/ASME Trans Mechatron. 2010;15(6):862–870.

[46] Liu H, Selvaggio M, Ferrentino P, Moccia R, Pirozzi S, Bracale U, Ficuciello F. The MUSHA hand II: A multifunctional hand for robot-assisted laparoscopic surgery. IEEE/ASME Trans Mechatron. 2020;26(1):393–404.

[47] Phan PT, Thai MT, Hoang TT, Lovell NH, Do TN. HFAM: Soft hydraulic filament artificial muscles for flexible robotic applications. IEEE Access. 2020;8:226637–226652.

[48] Davies J, Phan PT, Huang D, Hoang TT, Low H, Thai MT, Nguyen CC, Nicotra E, Lovell NH, Do TN. Hydraulically actuated soft tubular gripper. In: 2022 International Conference on Robotics and Automation (ICRA). Philadelphia (PA): IEEE; 2022. p. 6144–6150.

[49] Hirano J, Tanaka D, Watanabe T, Nakamura T. Development of delta robot driven by pneumatic artificial muscles. In: 2014 IEEE/ASME International Conference on Advanced Intelligent Mechatronics. Besançon (France): IEEE; 2014. p. 1400–1405.

[50] Atallah S, Sanchez A, Bianchi E, Larach S. Envisioning the future of colorectal surgery: Preclinical assessment and detailed description of an endoluminal robotic system (Colubris MX ELS). Tech Coloproctol. 2021;25(11):1199–1207. 34224035 10.1007/s10151-021-02481-0

[51] Joslin J, Biondich A, Walker K, Zanghi N. A comprehensive review of hirudiniasis: From historic uses of leeches to modern treatments of their bites. Wilderness Environ Med. 2017;28(4):355–361. 29030099 10.1016/j.wem.2017.08.002

[52] Mann KH. Leeches (Hirudinea): Their structure, physiology, ecology and embryology. Oxford: Pergamon Press; 2013.

[53] Bae JH, Yang DH, Lee JY, Soh JS, Lee S, Lee HS, Lee HJ, Park SH, Kim KJ, Ye BD, et al. Clinical outcomes of endoscopic submucosal dissection for large colorectal neoplasms: A comparison of protruding and laterally spreading tumors. Surg Endosc. 2016;30(4):1619–1628. 26169642 10.1007/s00464-015-4392-6

[54] Williamson T, Song SE. Robotic surgery techniques to improve traditional laparoscopy. JSLS. 2022;26(2):e2022.00002. 10.4293/JSLS.2022.00002 PMC9135605 35655469

[55] Alazmani A, Hood A, Jayne D, Neville A, Culmer P. Quantitative assessment of colorectal morphology: Implications for robotic colonoscopy. Med Eng Phys. 2016;38(2):148–154. 26762775 10.1016/j.medengphy.2015.11.018

[56] Webster RJ III, Jones BA. Design and kinematic modeling of constant curvature continuum robots: A review. Int J Robot Res. 2010;29(13):1661–1683.

[57] Traeger MF, Roppenecker DB, Coy J, Fiolka A, Wilhelm D, Scheider A, Meining A, Feussner H, Lueth TC. Forces in minimally invasive surgery: Reliable manipulation of gastric mucosa and the sigmoid colon. In: 2014 IEEE international conference on robotics and biomimetics (ROBIO 2014). Bali (Indonesia): IEEE; 2014. p. 408–412.

[58] Mustaza SM, Elsayed Y, Lekakou C, Saaj C, Fras J. Dynamic modeling of fiber-reinforced soft manipulator: A visco-hyperelastic material-based continuum mechanics approach. Soft Robot. 2019;6(3):305–317. 30917093 10.1089/soro.2018.0032

[59] Pandav K, Te AG, Tomer N, Nair SS, Tewari AK. Leveraging 5G technology for robotic surgery and cancer care. Cancer Rep. 2022;5(8): Article e1595. 10.1002/cnr2.1595 PMC9351674 35266317

[60] Cetinsaya B, Gromski MA, Lee S, Xia Z, Demirel D, Halic T, Bayrak C, Jackson C, De S, Hegde S, et al. A task and performance analysis of endoscopic submucosal dissection (ESD) surgery. Surg Endosc. 2019;33(2):592–606. 30128824 10.1007/s00464-018-6379-6 PMC6344246

[61] Gao H, Yang X, Xiao X, Zhu X, Zhang T, Hou C, Liu H, Meng MQH, Sun L, Zuo X, et al. Transendoscopic flexible parallel continuum robotic mechanism for bimanual endoscopic submucosal dissection. Int J Robot Res. 2024;43(3):281–304.

[62] Lau KC, Leung EYY, Chiu PWY, Yam Y, Lau JYW, Poon CCY. A flexible surgical robotic system for removal of early-stage gastrointestinal cancers by endoscopic submucosal dissection. IEEE Trans Industr Inform. 2016;12(6):2365–2374.

[63] Yang Y, Li J, Kong K, Wang S. Design of a dexterous robotic surgical instrument with a novel bending mechanism. Int J Med Robot. 2022;18(1): Article e2334. 34551453 10.1002/rcs.2334

[64] Gonzalez EC, Roetzheim RG, Ferrante JM, Campbell R. Predictors of proximal vs. distal colorectal cancers. Dis Colon Rectum. 2001;44(2):251–258. 11227943 10.1007/BF02234301

[65] Hwang M, Kwon DS. K-FLEX: A flexible robotic platform for scar-free endoscopic surgery. Int J Med Robot. 2020;16(2): Article e2078. 31945797 10.1002/rcs.2078

