# Supplementary material for: Development of a Bioinspired Soft Robotic System for Teleoperated Endoscopic Surgery
Source: Cyborg Bionic Syst. 2025 Jun 12;6:0289. doi: 10.34133/cbsystems.0289 (PMC12159415; doi:10.34133/cbsystems.0289)
Supplement: Supplementary 1 — Figs. S1 to S7 Tables S1 and S2 Movies S1 to S5 [file cbsystems.0289.f1.zip › Revised_Supporting Information.docx]

SUPPORTING INFORMATION

**Title**

Development of a Bio-inspired Soft Robotic System for Teleoperated Endoscopic Surgery

**Authors**

Kefan Zhu^1^, Chi Cong Nguyen^1^, Bibhu Sharma^1^, Phuoc Thien Phan^1^, Trung Thien Hoang^1^, James Davies^1^, Adrienne Ji^1^, Emanuele Nicotra^1^, Jingjing Wan^1^, Patrick Pruscino^1^, Sumeet Walia^2^, Tat Thang Vo-Doan^3^, Soo Jay Phee^4^, Shing Wong^5^, Nigel H. Lovell^1,6^, Thanh Nho Do^1,6*^

**Affiliations**

^1^ Graduate School of Biomedical Engineering, UNSW, Kensington Campus, Sydney, NSW 2052, Australia.

^2^ Centre for Opto-electronic Materials and Sensors, School of Engineering, RMIT University, Melbourne, VIC 3000, Australia.

^3^ School of Mechanical & Mining Engineering, The University of Queensland, St Lucia, Queensland, 4072 Australia

^4^ School of Mechanical and Aerospace Engineering, Nanyang Technological University, 50 Nanyang Avenue, Singapore 639798

^5^ School of Clinical Medicine, UNSW Sydney and Prince of Wales Hospital, Randwick, Sydney, NSW, 2031 Australia

^6^Tyree Institute of Health Engineering (IHealthE), Faculty of Engineering, UNSW, Sydney, NSW 2052, Australia.

^*^Address correspondence to: tn.do@unsw.edu.

**Table S1.** List of acronyms and abbreviations.

| CRC | Colorectal cancer |
| --- | --- |
| EMR | endoscopic mucosal resection |
| ESD | endoscopic submucosal dissection |
| DOFs | degrees of freedom |
| LS | laparoscopic surgery |
| HFAMs | hydraulic filament artificial muscles |
| TSGS | three-jaw soft grasper system |
| NOTES | natural orifice transluminal endoscopic surgery |
| SLA | stereolithography |
| FDM | fused deposition modelling |
| EM | Electromagnetic |
| EMI | electromagnetic interference |
| DEA | dielectric elastomer actuator |

**Table S2.** Symbol of mathematical model.

| $L_{m}$ | The total length of 0.8 mm muscle |
| --- | --- |
| $\gamma$ | The opening angle of grasper. |
| $\varphi$ | The wrapping angle of muscle. |
| $h$ | The total length of the jaw. |
| $R_{i}$ | The radius of the base of grasper. |
| $h_{0}$ | The distance between a connection of the base and the starting point of the muscle. |
| $h_{m}$ | The distance between the intersection points of two jaws and the connection of the base. |
| $h_{i}$ | The distance between the connection of the base and *i* ^th^ segment of muscle. |
| $x_{i}$ | The length of the *i* ^th^ segment of muscle. |
| $n$ | The number of segments of muscle that wrap around jaws. |
| $V_{i}$ | The input volume of *i* ^th^ syringe. |
| ${LS}_{i}$ | The distance between the centroids of the controller and each piston of the syringe. |
| $k_{c}$ | The stiffness of the constraining coil. |
| $k_{r}$ | The instantaneous spring constant of the silicone tubing. |
| $L_{i}$ | The elongation length of *i* ^th^ muscle. |
| $L_{o}$ | The initial length of the muscle. |
| *E* | The Young’s modulus of the silicone tubing. |
| $A_{tube}$ | The cross-section area of the silicone tubing. |
| *S* | The radius of curvature of the manipulator. |
| *m* | The array radius of muscles. |
| $L_{1}$ | The elongation length of the first muscle. |
| $L_{2}$ | The elongation length of the second muscle. |
| $L_{3}$ | The elongation length of the third muscle. |
| $\alpha$ | The rotating angle of the plane containing the manipulator. |
| $\beta$ | The radians of curvature. |
| $X_{e}$ | The position of the end-effector of the manipulator in the x-axis |
| $Y_{e}$ | The position of the end-effector of the manipulator in the y-axis |
| $Z_{e}$ | The position of the end-effector of the manipulator in the axis |

**Supplementary Note 1. Materials and Fabrication Methods**

The fabrication steps of the grasper are shown in Figure 2B. Three sharp needles go through and stick to the holes, and pointed projections are designed to enhance gripping capability by penetrating or securing the manipulated object. The back of the jaw curves inward at a right angle, forming a groove structure that assists in retaining the artificial muscles within the jaw. Three jaws are symmetrically hooked on the base of the grasper mechanism. The jaws and base of the grasper are constructed utilizing a Stereolithography (SLA) 3D printing technique, the material used for the grasper's fabrication is biocompatible resin (Formlabs, BioMed Clear Resin), selected for its Hardness 78D mechanical properties and biocompatibility, ensuring safety and functionality during surgical operations. The Needles were cut from 0.3 mm insulin syringes (BD Medical-Diabetes Care, France). Opening the jaws, attach the elastic band on the elastic band hook and fix another end inside of the base. Test whether the grasper can rebound with the jaw if it is manually closed. The muscle sticks out from the center axis of the grasper and wraps around the jaws. and attached to the one tip of a jaw with superglue. The muscle is constraining jaws to close. The actuator muscle is constructed from 0.6 mm STHT-C-020-0 silicon tube (Sani-Tech West, Camarillo, CA, USA), 0.8 mm helical coils (McMaster-Carr Supply Co., Elmhurst, IL, USA), and BD Luer-Lok™ 1-mL syringes, based on hydraulic filament artificial muscles. The working principle of the actuator muscle is as follows: in its resting state, the grasper is constrained by muscle and maintains a close state. The muscle is elongated due to the pressurization achieved by pushing the syringe. The constraint area is extended, and the jaws start opening. Upon releasing the syringe's piston, the stored potential energy within the microtubules and helical coils triggers the contraction of the muscles, thereby gripping tissue. The muscle sticks out from the center axis of the grasper and is wrapped around the jaws.

To manipulate the position of the grasper, the manipulator is designed with three muscles, each comprising a 2 mm STHT-C-030-0 silicone tube (Sani-Tech West, Camarillo, CA, USA), encased within a 2.5 mm helical coil and arranged in a triangular configuration spaced 120° apart. The muscles are combined using thin-cut heat shrink tubing placed on their surface. Each silicone tube is connected to a transmission tube, facilitating the transfer of hydraulic pressure from the syringes to the silicone tubes. Upon the application of hydraulic pressure, the manipulator is capable of elongation, omnidirectional bending, and rotation. The top of the manipulator is secured with white string to connect the silicone tubes and helical coil, while the bottom is fixed and covered with heat shrink to prevent the silicone tubes from detaching. To address the issue of non-uniform gap formation between heat shrink segments during elongation, which causes unpredictable bending motions, the manipulator is pre-elongated by 50% and then coated in EcoFlex 00-30 silicone (Smooth-On, Inc, USA). This coating ensures that the heat shrink moves uniformly during elongation, and 50% pre-elongation can prevent the silicone from limiting the manipulator's elongation capabilities. The biocompatible silicone protective layer enables the manipulator to operate safely within the colon.

The main body of the master device is fabricated using 3D printing technology. As shown in Figure 3a, supporting information, the grey components are produced with an SLA printer. In contrast, the white components are fabricated using a fused deposition modeling (FDM) printer with tough polylactic acid (PLA) material. Each pair of magnetic ball joints is connected using a 150mm M4 threaded rod. 4×10×4 mm roller bearings (Plaig Bearings, AUS) facilitate smooth motion between the crank and slider. The sliders are guided by two parallel steel rods, each with a diameter of 4mm, which are anchored through designated holes in the base. Linear motion of the sliders is enabled by 4×8×12 mm linear bearings (Plaig Bearings, AUS) positioned between the steel rods and sliders, allowing for unidirectional translation with minimal friction.

The cost and difficulty of sterilization are critical considerations for a surgical device. The materials of the surgical instruments are mainly helical coils and medical silicone tubes, which are common. They can be disposable because of the low price. Moreover, the motorless configuration significantly reduces overall cost. Autoclaving or ethylene oxide gas can be used to sterilize surgical instruments. Silicone tubes, biocompatible metal alloys, biocompatible resin, and plastic transmission tubes can all withstand at least one cycle of these methods. In addition, this device is motorless and completely sealed, so there is no need to worry about moisture and debris entering the instrument's interior and damaging the electronic components during the cleaning and sterilization procedure.

**Supplementary Note 2. Force detection of master-slave device**

To evaluate the force efficiency of the system, we compared the inward force at the end of the device with the inward force exerted on the three syringes. A 6-axis force sensor Nano 17 (ATI, USA) with a handle was mounted at the end of the master device. The operator manipulated the end-effector to the same point via the handle while the force sensor recorded the operational force in real time (Figure S1A). Three pressure sensors were connected to the syringes, and the force exerted on each syringe was calculated by multiplying the measured pressure by the inner surface area of the syringe. After conducting eight trials, the results were averaged and plotted in Figure S1B. The input and output forces, averaging over eight experiments, showed a force ratio (output/input) of 1.45.

Supplementary Note 3. Mapping of master-slave device

The mapping between the master device's end-effector and the slave device's manipulator is defined by the relationship between key points in the system: *PP, PL,* and *PB*, which represent the connection points of the end-effector, the rod, and the base, respectively. From the mathematical diagram **(Figure S1C)**, the relationship between points *PP* and *PB* can be found in the following equation:

| $\left\{ \left. {PD}_{i} \right\}+\left\{ \left. I_{i} \right\} \right. \right.=\left\{ \left. {PB}_{i} \right\} \right.+\left\{ \left. D_{i} \right\} \right.+\left\{ \left. I_{i} \right\} \right.= \left\{ \left. {PP}_{i} \right\} \right.$ | (5) |
| --- | --- |

where $D_{i}$ and $I_{i}$ is the vectors of the crank and rod, *i* represents the number of parts ($i=1, 2, 3$), and *I* denotes the rod length. Since the base of the master device is fixed, the value of $\left\{ \left. {PB}_{i} \right\} \right.$ is defined as constant. The vectors $\left\{ \left. {PD}_{i} \right\} \right.$ are dependent on joint angle $\theta_{i}$, which is between the crank and base*.* The master device's end-effector is constrained to motion within the Cartesian space *(x, y, z),* because the master device can only move in *XYZ* translation. we can get the $\left\{ \left. {PP}_{i} \right\} \right.$values. Then, following the below equation, we can find the $\theta_{i}$ through end effector position*.* Furthermore, the $\theta_{i}$ is constrained between 20 to 90 degrees, the working space of the master device can be calculated from the equation in MATLAB (Mathworks, Inc., USA) **(Figure S2A)**.

| $\left\{ \left. {PB}_{1} \right\} \right.=\left\{ \left. \begin{matrix} 0 \\ -B \\ 0 \end{matrix} \right\} \right. \left\{ \left. D_{1} \right\} \right.=\left\{ \left. \begin{matrix} 0 \\ -Dcos\left( \theta_{1} \right) \\ -Dsin\left( \theta_{1} \right) \end{matrix} \right\} \right. \left\{ \left. {PP}_{1} \right\} \right.=\left\{ \left. \begin{matrix} x \\ y-P \\ z \end{matrix} \right\} \right.$ | (6) |
| --- | --- |
| $\left\{ \left. I_{1} \right\} \right.=\left\{ \left. \begin{matrix} x \\ y+Dcos\left( \theta_{1} \right)+B-P \\ z+Dsin\left( \theta_{1} \right) \end{matrix} \right\} \right.$ | (7) |
| $\left\{ \left. {PB}_{2} \right\} \right.=\left\{ \left. \begin{matrix} \frac{B}{\sqrt{3}} \\ \frac{B}{2} \\ 0 \end{matrix} \right\} \right. \left\{ \left. L_{2} \right\} \right.=\left\{ \left. \begin{matrix} \frac{\sqrt{3}}{2}Dcos\left( \theta_{2} \right) \\ \frac{1}{2}Dcos\left( \theta_{2} \right) \\ -Dsin\left( \theta_{2} \right) \end{matrix} \right\} \right. \left\{ \left. {PP}_{2} \right\} \right.=\left\{ \left. \begin{matrix} x+\frac{P}{\sqrt{3}} \\ y+\frac{P}{2} \\ z \end{matrix} \right\} \right.$ | (8) |
| $\left\{ \left. I_{2} \right\} \right.=\left\{ \left. \begin{matrix} x+\frac{P}{\sqrt{3}}-\frac{B}{\sqrt{3}}-\frac{\sqrt{3}}{2}Dcos\left( \theta_{2} \right) \\ y+\frac{P}{2}-\frac{1}{2}Dcos\left( \theta_{2} \right)-\frac{B}{2} \\ z+Dsin\left( \theta_{2} \right) \end{matrix} \right\} \right.$ | (9) |
| $\left\{ \left. {PB}_{3} \right\} \right.=\left\{ \left. \begin{matrix} -\frac{B}{\sqrt{3}} \\ \frac{B}{2} \\ 0 \end{matrix} \right\} \right. \left\{ \left. L_{3} \right\} \right.=\left\{ \left. \begin{matrix} -\frac{\sqrt{3}}{2}Dcos\left( \theta_{3} \right) \\ \frac{1}{2}Dcos\left( \theta_{3} \right) \\ -Dsin\left( \theta_{3} \right) \end{matrix} \right\} \right. \left\{ \left. {PP}_{3} \right\} \right.=\left\{ \left. \begin{matrix} x-\frac{P}{\sqrt{3}} \\ y+\frac{P}{2} \\ z \end{matrix} \right\} \right.$ | (10) |
| $\left\{ \left. I_{3} \right\} \right.=\left\{ \left. \begin{matrix} x-\frac{P}{\sqrt{3}}+\frac{\sqrt{3}}{2}Dcos\left( \theta_{3} \right)+\frac{B}{\sqrt{3}} \\ y+\frac{P}{2}-\frac{1}{2}Dcos\left( \theta_{3} \right)-\frac{B}{2} \\ z+Dsin\left( \theta_{3} \right) \end{matrix} \right\} \right.$ | (11) |
| $\left\{ \left. I_{i} \right\} \right.^{2}={\vert\left\vert\left\{ \left. {PP}_{i} \right\} \right.-\left\{ \left. {PB}_{i} \right\} \right.-\left\{ \left. D_{i} \right\} \right. \right\vert\vert}^{2}=I^{2}$ | (12) |

Where *B* is the length of the base, and *P* is the length of the end-effector. After figuring out the $\theta_{i}$, the relationship between piston position of syringe *LS* and position of end-effector of the master device *(x, y, z)* can be found in Scotch Yoke mechanisms **(Figure S2B, Supporting Information)**.

| $\Delta{LS}_{i}=tan( 110-{\Delta\theta}_{i}(x,y,z))Lr$ | (13) |
| --- | --- |

Where *Lr* is the length from the piston to the connection between the slider and crank. We know the inner radius of the syringe chamber is 2.23mm, and the input volume *V* can be described as

| $V_{i}(\Delta{LS}_{i})={2.23}^{2}\pi\Delta{LS}_{i}tan( 110-{\Delta\theta}_{i})$ | (14) |
| --- | --- |

Then, the below equation can be used to get the elongation length of muscle *L* from input volume based on the stiffness of the constraining coil $k_{c}$, the instantaneous spring constant of the silicone tubing $k_{r}$*,* the initial length of muscles $L_{o}$, the Young’s modulus *E* and cross-section area $A_{tube}$ of the silicone tubing.

| $L_{i}(V_{i})=\frac{2(k_{c}L_{o}+EA_{tube})}{L_{o}A_{tube}\left( k_{c}+k_{r} \right)-2V_{i}k_{c}}$ | (15) |
| --- | --- |

The final position of the manipulator’s end-effector$\left\{ \left. \begin{matrix} X_{e} & Y_{e} & Z_{e} \end{matrix} \right\} \right.^{T}$ is derived using the continuum robot kinematics, with the radius of curvature of manipulator *S*, array radius of muscles *m*, rotating angle of the plane of the manipulator $\alpha$,radians of manipulator curvature $\beta$. $L_{1}$, $L_{2}$, $L_{3}$ represents elongation length of three different muscles. The final 30 mm diameter working space of the end-effector of manipulator is shown in **Figure S2C (Supporting Information)**, which is enough for ESD surgery.

| $S= \frac{m\left( 3L_{o}+L_{1}+L_{2}+L_{3} \right)}{2\sqrt{{L_{1}}^{2}+{L_{2}}^{2}+{L_{3}}^{2}-L_{1}L_{2}-L_{2}L_{3}-L_{3}L_{1}}}$ | (16) |
| --- | --- |
| $\alpha=\tan^{-1} \left[ \frac{\sqrt{3}\left( L_{3}+L_{2}-2L_{1} \right)}{3\left( L_{2}-L_{3} \right)} \right]$ | (17) |
| $\beta=\frac{2\sqrt{{L_{1}}^{2}+{L_{2}}^{2}+{L_{3}}^{2}-L_{1}L_{2}-L_{2}L_{3}-L_{3}L_{1}}}{3m}$ | (18) |
| $\left\{ \left. \begin{matrix} X_{e} \\ Y_{e} \\ Z_{e} \end{matrix} \right\} \right.=\left\{ \left. \begin{matrix} S\left( 1-cos\beta\right)cos\alpha\\ S\left( 1-cos\beta\right)sin\alpha\\ Ssin\beta\end{matrix} \right\} \right.$ | (19) |


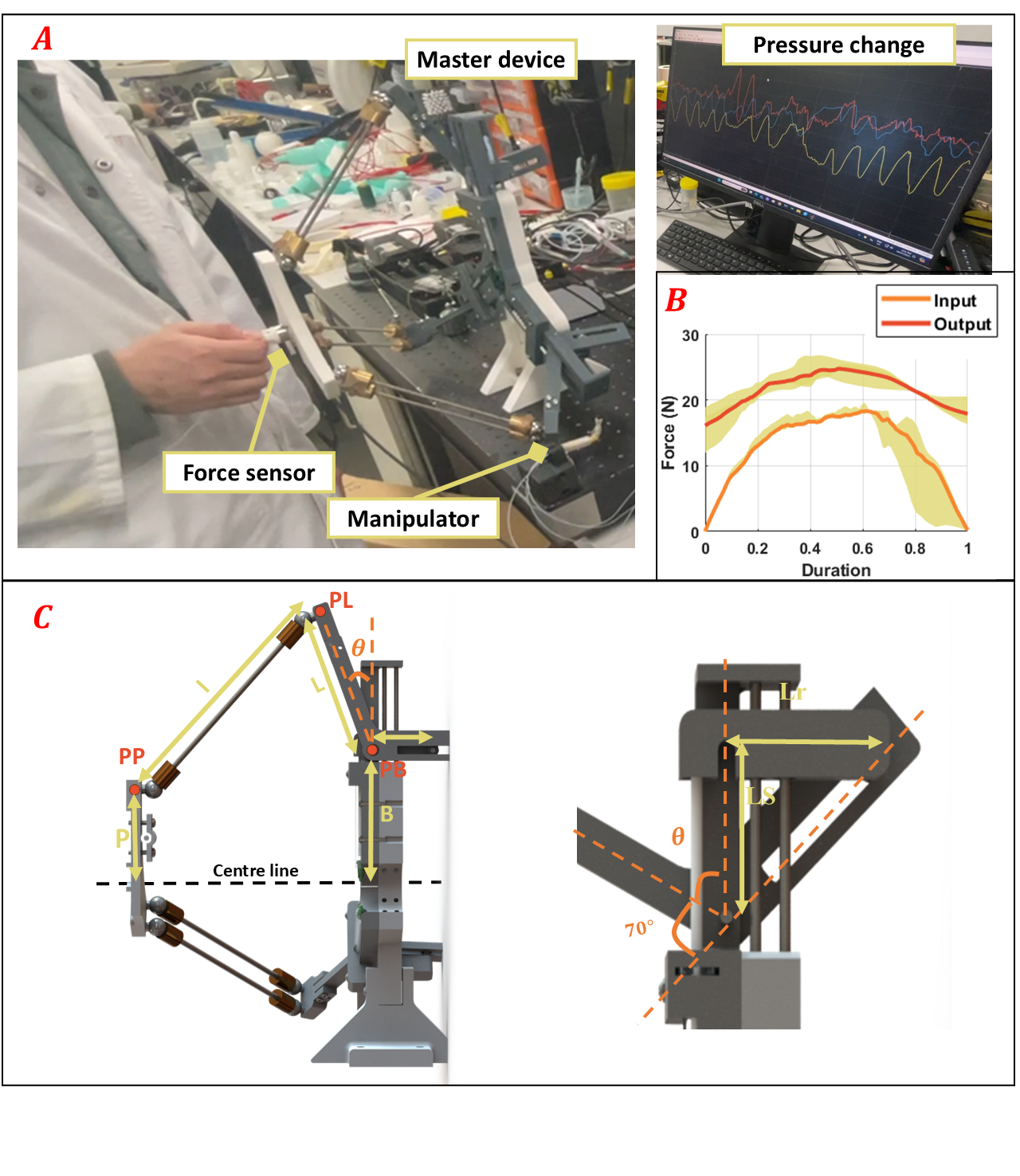


**Figure S1.** Force Detection and Model Analysis. (A) Experiment setup for force detection. (B) Experiment results for force detection. (C) Mathematical model of the master device and Scotch Yoke mechanism.


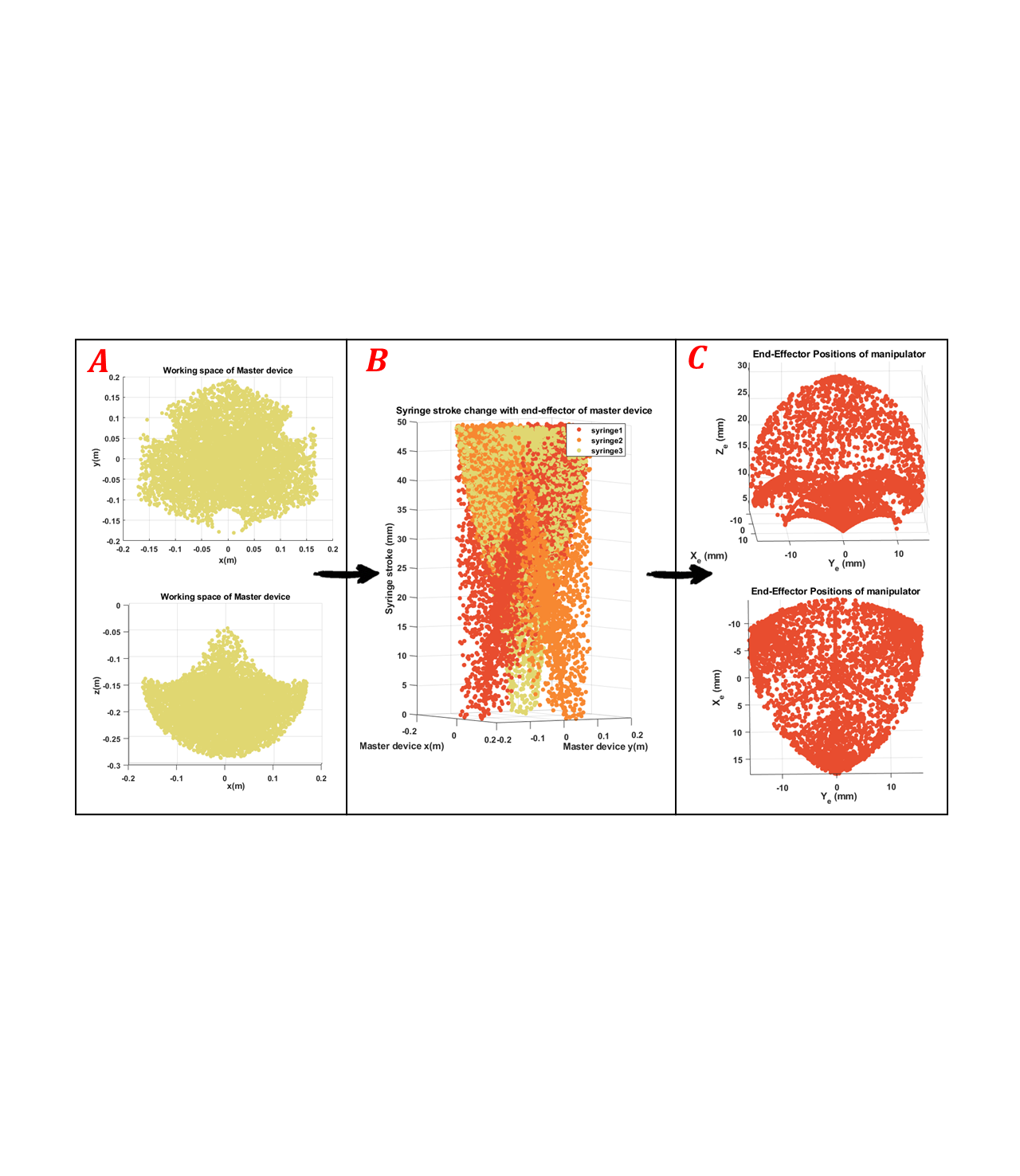


**Figure S2.** Theoretical Working Space and Stroke Analysis. (A) The theoretical working space of the master device is solved by the mathematical model. (B) Theoretical syringe stroke changes, based on the movement of the end-effector of the master device in the XY plane. (C) Theoretical working space of manipulator.

**Supplementary Note 4. Experimental setups for characterizations of the TSGS**


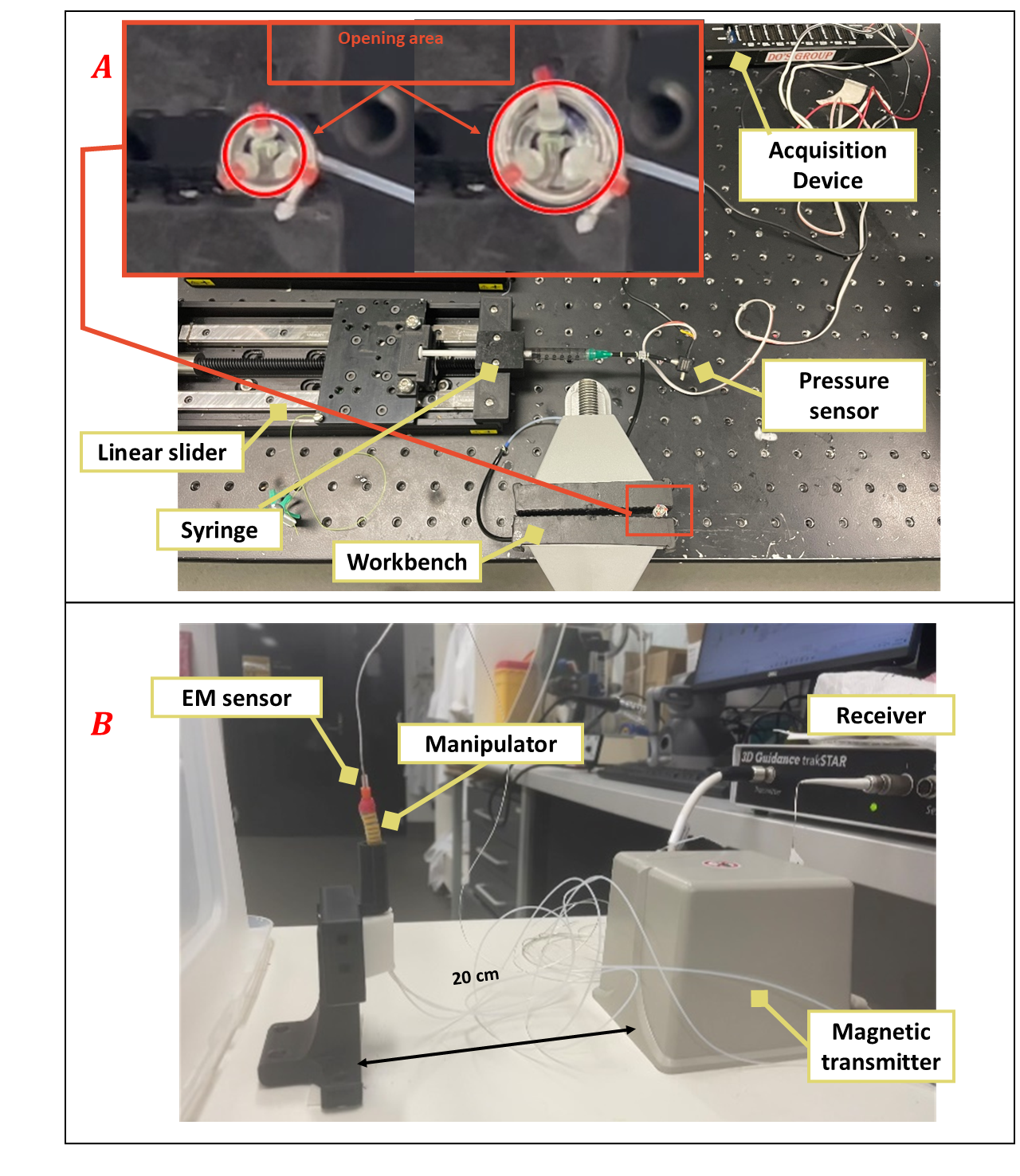


**Figure S3.** Analysis of Grasper Opening Area and Manipulator Working Space. (A) Experiment setup for Opening area by imaging analysis and comparing with the mathematical model. (B) Experiment setup for the working space of the manipulator.


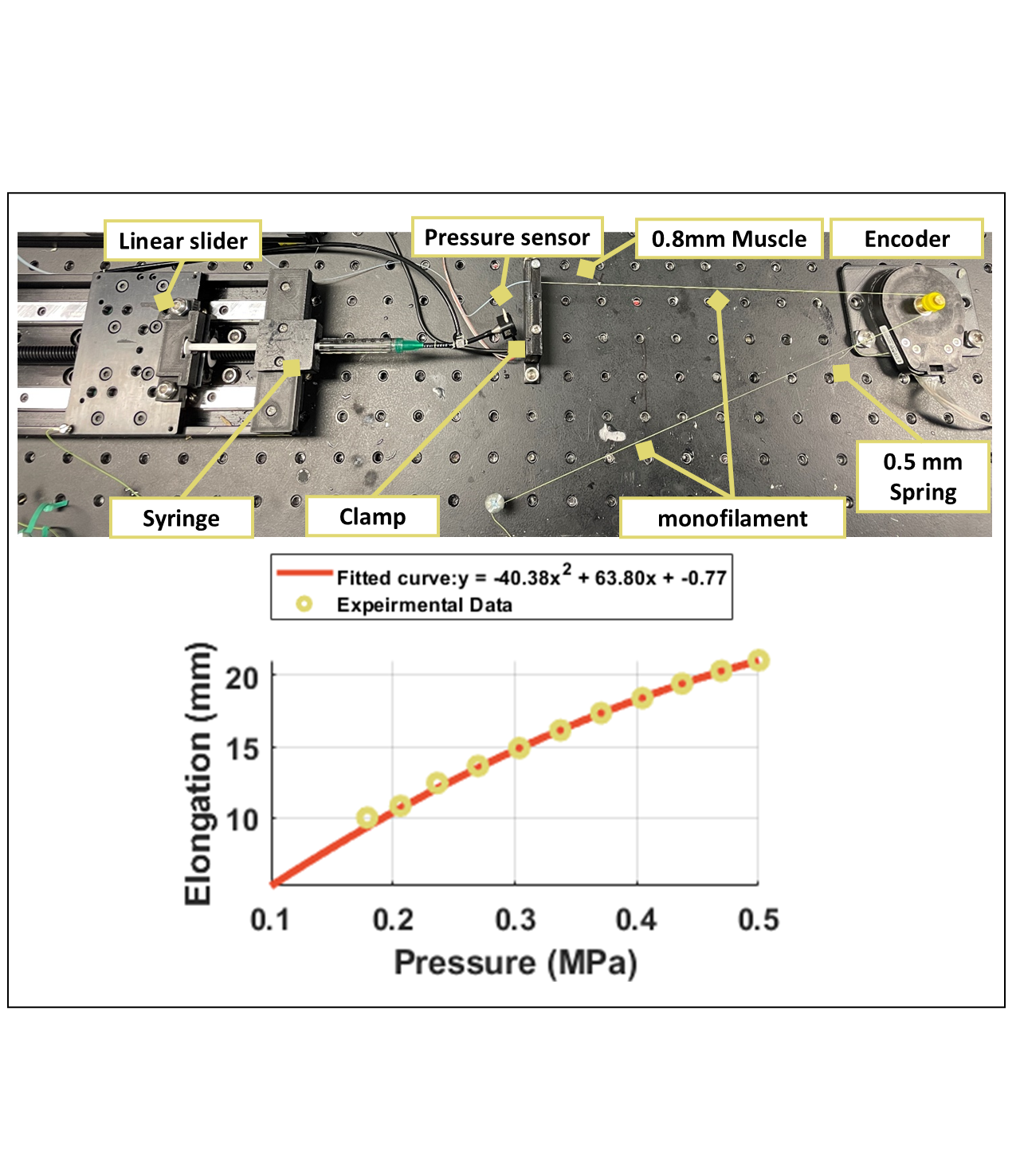


**Figure S4.** Experiment setup and results for 0.8 mm artificial muscle characterization.


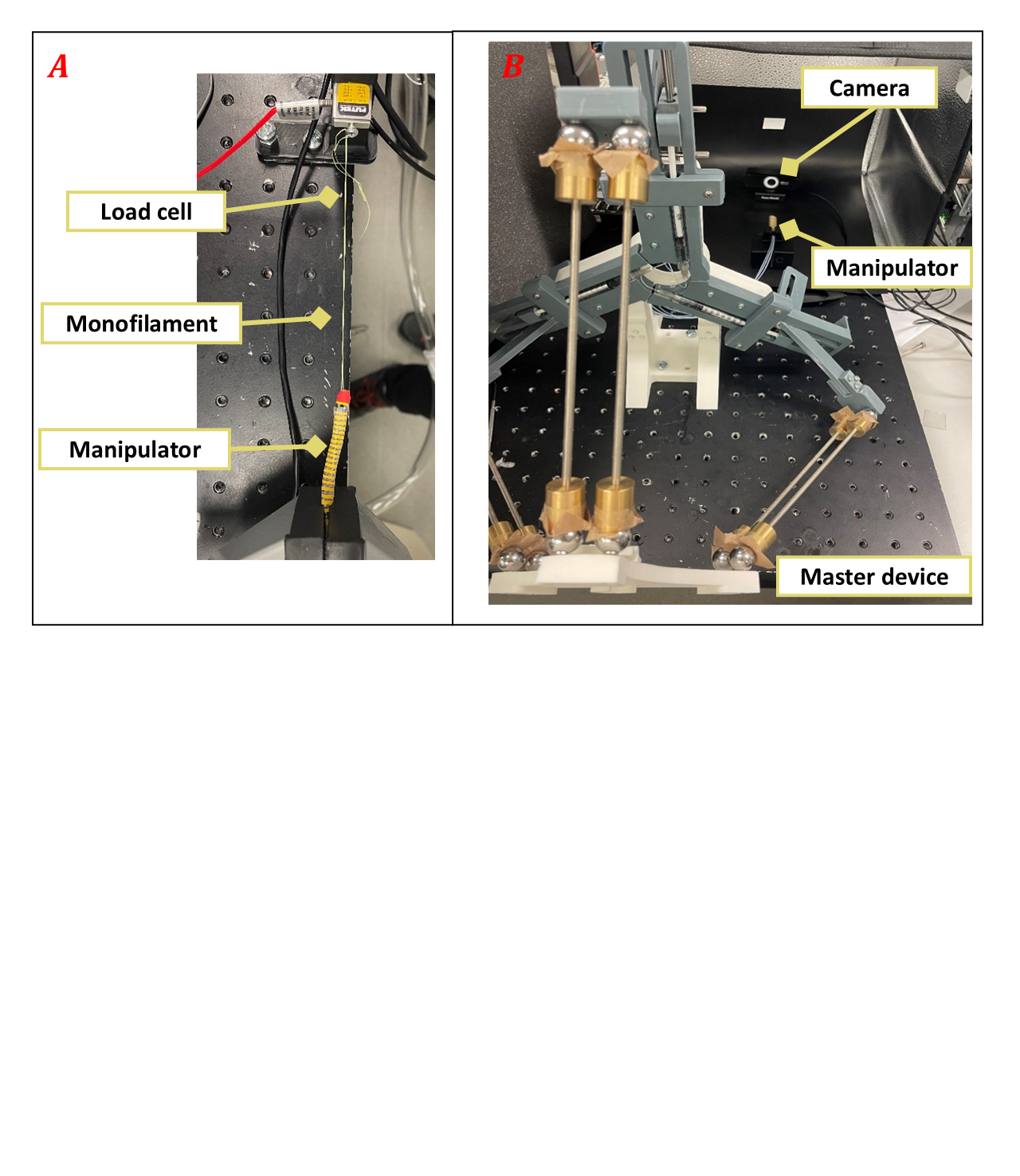


**Figure S5.** Pulling Force and Steering Characterization. (A) Experiment setup for pulling force characterization. (B) Experiment setup for steering of the master-slave device.

**Supplementary Note 5. Response Time**

The response time of the TSGS is defined as the duration required for energy transfer from the human hand to the surgical instrument. Therefore, two force sensors (FUTEK, USA) were placed on the handle of the master device and the other on the surgical instrument. These sensors captured the force onset times, enabling the response time calculation by measuring the temporal difference between the initial human-applied force and the corresponding instrument response. Synchronized data acquisition at 500 Hz sampling frequency enabled precise detection of force onset timestamps, with the response time calculated as the temporal difference between these two events.

A total of 11 trials were conducted under the same experimental conditions. The top graph illustrates the response time for each cycle across these trials, demonstrating variations between approximately 0.05 and 0.11 seconds. The mean response time was calculated as 0.0895 seconds, with a standard deviation of 0.0227 seconds. Variations in response time may be attributed to air bubbles within the transmission tube. Air bubbles entrapped within the silicone transmission tubes introduce compressibility to the incompressible fluidic system. During pressurization, energy dissipation occurs as bubbles absorb and slowly release pressure, elongating the force propagation time. Internal resistance at sliding interfaces generates counteracting forces that impede smooth energy transfer. Frictional losses are particularly pronounced in components with high surface contact, such as the Scotch Yoke mechanism described in the original design. These limitations can be improved by using a vacuum-assisted degassing method and medical-grade lubricants.

**
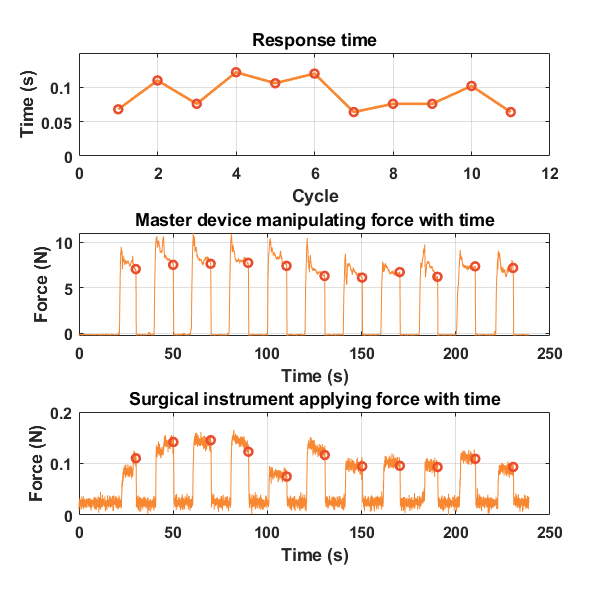
**

**Figure S6.** Measured response time over 11 consecutive force‐manipulation cycles in the TSGS (top). The middle plot depicts the time of the master device’s applied force, while the bottom plot shows the corresponding time at the surgical instrument.

**Supplementary Note 6. Durability**

To test the TSGS's durability, the system was operated continuously for 40 minutes under 6 seconds of circle time. Each cycle consisted of 4 seconds of continuous motion followed by 2 seconds of static positioning at a predefined location. To accurately track the end effector's position, two cameras were placed orthogonally, and a maker was placed on the end effector position for further analysis. One captures the front view, and the other captures the surgical instrument's side view to gather the Cartesian coordinates of the end effector's position. A marker was attached to the end effector to define the end effector position for subsequent image analysis. A comparative study of initial versus final operational periods revealed significant positional drift: X-axis displacement decreased by 34.2%, while Y-axis variation remained minimal at 0.9% under the highest pressure. The positive proportional relationship between applied pressure and position maintenance can be seen from the curve trend in the figure. The potential problem could be inside the manipulator, the three silicone tubes are affected by each other. Because silicone’s low Young’s modulus permits radial expansion ratios, it exhibits hyperplastic behavior that facilitates fluid power transmission and promotes energy transfer among adjacent tubes. The tube with lower internal pressure may be deformed by higher-pressure tubes, leading to undesirable displacement.


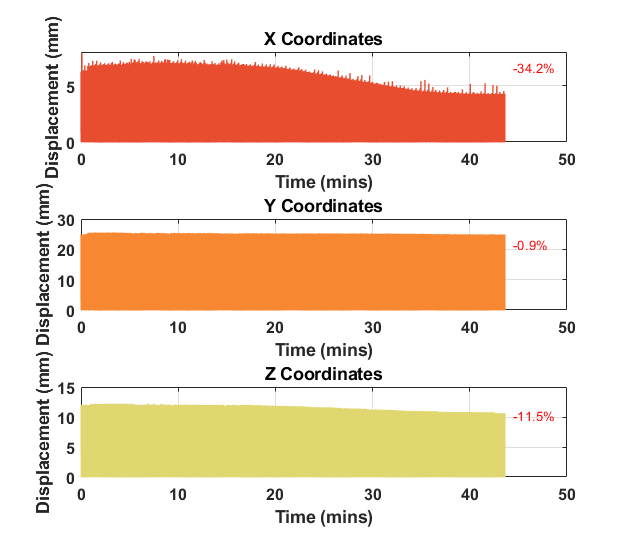


**Figure S7.** Durability test results show the TSGS manipulator’s X, Y, and Z displacements over 40 minutes of continuous operation.

**Supplementary Note 7. Legends of Supplementary Movies**

**Video S1**. The imaging analysis of the opening area.

**Video S2**. Manipulating the grasper to grip the tumor model by the master device.

**Video S3**. The grasping procedure in the in-vitro experiment.

**Video S4**. The grasping procedure in the ex-vivo experiment.

**Video S5**. The grasping and cauterizing procedure in ex-vivo experiment.
